# Improving Aqueous Solubility of Natural Antioxidant Mangiferin through Glycosylation by Maltogenic Amylase from *Parageobacillus galactosidasius* DSM 18751

**DOI:** 10.3390/antiox10111817

**Published:** 2021-11-16

**Authors:** Jiumn-Yih Wu, Hsiou-Yu Ding, Tzi-Yuan Wang, Yu-Li Tsai, Huei-Ju Ting, Te-Sheng Chang

**Affiliations:** 1 Department of Food Science, National Quemoy University, Kinmen County 892, Taiwan; wujy@nqu.edu.tw; 2 Department of Cosmetic Science, Chia Nan University of Pharmacy and Science, No. 60 Erh-Jen Rd., Sec. 1, Jen-Te District, Tainan 71710, Taiwan; ding8896@gmail.com; 3 Biodiversity Research Center, Academia Sinica, Taipei 11529, Taiwan; tziyuan@gmail.com; 4 Department of Biological Sciences and Technology, National University of Tainan, Tainan 70005, Taiwan; aa0920281529@gmail.com (Y.-L.T.); hting@mail.nutn.edu.tw (H.-J.T.)

**Keywords:** mangiferin, maltogenic amylase, glycosylation, glucoside, *Parageobacillus galactosidasius*

## Abstract

Mangiferin is a natural antioxidant *C*-glucosidic xanthone originally isolated from the *Mangifera indica* (mango) plant. Mangiferin exhibits a wide range of pharmaceutical activities. However, mangiferin’s poor solubility limits its applications. To resolve this limitation of mangiferin, enzymatic glycosylation of mangiferin to produce more soluble mangiferin glucosides was evaluated. Herein, the recombinant maltogenic amylase (MA; E.C. 3.2.1.133) from a thermophile *Parageobacillus galactosidasius* DSM 18751^T^ (*Pg*MA) was cloned into *Escherichia coli* BL21 (DE3) via the expression plasmid pET-Duet-1. The recombinant *Pg*MA was purified via Ni^2+^ affinity chromatography. To evaluate its transglycosylation activity, 17 molecules, including mangiferin (as sugar acceptors), belonging to triterpenoids, saponins, flavonoids, and polyphenol glycosides, were assayed with β-CD (as the sugar donor). The results showed that puerarin and mangiferin are suitable sugar acceptors in the transglycosylation reaction. The glycosylation products from mangiferin by *Pg*MA were isolated using preparative high-performance liquid chromatography. Their chemical structures were glucosyl-*α*-(1→6)-mangiferin and maltosyl-*α*-(1→6)-mangiferin, determined by mass and nucleic magnetic resonance spectral analysis. The newly identified maltosyl-*α*-(1→6)-mangiferin showed 5500-fold higher aqueous solubility than that of mangiferin, and both mangiferin glucosides exhibited similar 1,1-diphenyl-2-picrylhydrazyl free radical scavenging activities compared to mangiferin. *Pg*MA is the first MA with glycosylation activity toward mangiferin, meaning mangiferin glucosides have potential future applications.

## 1. Introduction

Mangiferin is a natural *C*-glucosidic xanthone originally isolated from the *Mangifera indica* (mango) plant. Mangiferin has been reported to possess diverse health-promoting activities, such as antioxidant [1,2], anticancer [3,4], anti-inflammatory [5], and anti-osteoarthritis pain activities [6], allowing it to prevent memory impairment [7], neurodegeneration [8], and organ fibrosis [9]. Furthermore, it offers protection from the deleterious effects of heavy metals [10]. However, the pharmacological use of mangiferin is restricted owing to its poor solubility and low bioavailability [11,12]. As the glycosylates of small molecules have been proven to have better aqueous solubility and bioavailability than the original molecules [13,14], the glycosylation of mangiferin should be further improved for better usage.

Glycosylation of molecules can be achieved using chemical or enzymatic methods; however, enzymatic glycosylation using glycosyltransferases (GTs) and glycoside hydrolases (GHs) offers more advantages than chemical methods [15]. Moreover, GHs use cheaper sugars, such as starch, maltodextrin, maltose, and sucrose, as donors during glycosylation [16], whereas GTs use expensive uridine diphosphate-glucose (UDP-G). Therefore, GHs are preferred for the bioindustrial production of glycosylated molecules. According to the carbohydrate-activating enzyme (CAZy) database, a classification of GH in families based on amino acid sequence similarities and 117 GH families has been discovered to date [17].

Maltogenic amylase (MA; E.C. 3.2.1.133) belongs to the GH13 gene family and hydrolyzes starch to produce maltose [17]. Some specific features of MA were further identified. First, MAs were found to prefer cyclodextrin (CD) to starch as a substrate, whereas typical amylases do not catalyze CD. The sugar preference is due to 130 unique residues at the N-terminal of the MA protein, which would help the enzyme form a dimer and greatly increase its catalytic activities toward CD [18,19]. Second, MAs are intracellular proteins, whereas typical amylases are extracellular proteins. Third, MAs exhibit the bifunctions of hydrolysis and transglycosylation activities [18,19,20,21,22,23,24,25,26,27,28], whereas typical amylases are rarely reported to have transglycosylation activities [18].

Based on the dual functions of hydrolysis and transglycosylation and dual recognition sites on both the *α*-1,4 and *α*-1,6 glycosidic bonds, MAs have also been used in the fine chemical industry to produce novel and branched oligosaccharides from liquefied starch [19,20,21,22,23,24,25,26,27,28]. MAs could be further used for the glycosylation of bioactive molecules to develop new drugs in the clinical chemistry field.

In addition to sugars, MAs can glycosylate small and/or bioactive molecules. MAs exhibit transglycosylation reactions in the presence of various acceptor molecules, such as glucose, maltose, and acarbose, by forming *α*-1,3, *α*-1,4, and *α*-1,6 glycosidic linkages [18,19,20,21,22,23,24,25,26,27,28]. MAs have been proven to glycosylate some small molecules, such as hydroquinone [29], caffeic acid [30], ascorbic acid [31], puerarin [32,33,34], genistin [35], neohesperidin [36], and naringin [37]. For example, the glycosylation of puerarin by two MAs, *Tf*MA from archaeon *Thermofilum pendens* [32] and *Bs*MA from *Bacillus stearothermophilus* [34], has been studied.

In the present study, a maltogenic amylase gene from *Parageobacillus galactosidasius* (*Pg*MA) was cloned into *Escherichia coli* BL21 (DE3) via the expression plasmid pET-Duet-1, and the expressed *Pg*MA was purified. The purified *Pg*MA was characterized and found to glycosylate mangiferin. The novel mangiferin glucosides were isolated for the characterization of both the chemical structures and compounds.

## 2. Materials and Methods

### 2.1. Reagents and Chemicals

A Ni^2+^ affinity column (10 i.d. × 50 mm, Ni Sepharose 6 Fast Flow) used for the purification of the recombinant MA was purchased from GE Healthcare (Chicago, IL, USA). Isopropyl β-d-1-thiogalactopyranoside (IPTG), 1,1-diphenyl-2-picrylhydrazine (DPPH), dimethyl sulfoxide (DMSO), and maltodextrin (dextrose equivalent 4.0–7.0) were bought from Sigma (St. Louis, MO, USA). *α*-CD, *β*-CD, *γ*-CD, soluble starch, and pullulan were purchased from Tokyo Chemical Industry Co., Ltd. (Tokyo, Japan). Restriction enzymes and DNA-modified enzymes were obtained from New England Biolabs (Ipswich, MA, USA). All kits for molecular cloning, including the Geno Plus Genomic DNA Extraction Midiprep System, Mini Plus Plasmid DNA Extraction System, Gel Advanced Gel Extraction Miniprep System, and Midi Plus Ultrapure Plasmid Extraction System, were purchased from Viogene (Taipei, Taiwan). Other reagents and solvents used are commercially available.

### 2.2. Strains and Plasmids

*P. galactosidasius* DSM 18751T (BCRC 80657) was obtained from the Bioresources Collection and Research Center (BCRC; Food Industry Research and Development Institute, Hsinchu, Taiwan). *E. coli* BL21 (DE3) and the expression plasmid pET-Duet-1 were obtained from Novagen Inc. (Madison, WI, USA).

### 2.3. Aligned Amino Acid Sequences

In total, 588 amino acids of *Pg*MA (OXB94089) and close-related BsMT (AAC46346) were aligned using Clustal W in MEGA X [38].

### 2.4. Construction of Expression Plasmids

*P. galactosidasius* DSM 18751T (BCRC 80657) was cultivated in accordance with the BCRC protocol. The genomic DNA of the bacterium was isolated using the Geno Plus Genomic DNA Extraction Midiprep System (Viogene) according to the manufacturer’s protocol. The target gene (*Pg*MA) was amplified from the genomic DNA with polymerase chain reaction (PCR). The primer set used in the PCR was as follows: forward 5′-gggggatccgttgaaagaagccatttatcatcg-3′ and reverse 5′-gggctcgagtcaattttctacttgatagaggag-3′, which contain BamHI and XhoI restriction sites (underlined mark) for cloning. The amplified DNA fragment (1.8 kb length) was cloned into the expression plasmid pET-Duet-1, named pETDuet-*Pg*MA, which was then transformed into *E. coli* BL21 (DE3) for the recombinant *Pg*MA.

### 2.5. Production and Purification of Recombinant PgMA in E. coli

The recombinant *E. coli* harboring the recombinant expression plasmid pETDuet-*Pg*MA was cultivated in Luria–Bertani (LB) medium containing 1% (*w*/*v*) tryptone and sodium chloride and 0.5% (*w*/*v*) yeast extract to the optical density at 560 nm (OD_560_) of 0.6 and then induced with 0.2 mM of IPTG. After further cultivation at 18 °C for 20 h, the cells were centrifuged at 4500× *g* and 4 °C for 20 min. The cell pellet was washed and spun down twice with 50 mM of phosphate buffer (PB) at pH 6.8 and then broken with sonication via a Branson S-450D Sonifier (Branson Ultrasonic Corp., Danbury, CT, USA). The sonication program was run for five cycles of 5 s on and 30 s off at 4 °C. The mixture was then centrifuged at 15,000× *g* and 4 °C for 20 min to remove the cell debris. A supernatant containing the recombinant *Pg*MA fused with a His-tag in its N-terminal was applied in an Ni^2+^ affinity column. The His-tag-fused *Pg*MA was washed with PB with 25 mM imidazole and eluted with PB containing 250 mM imidazole. The eluate was then concentrated and desalted through Macrosep 10 K centrifugal filters (Pall, Ann Arbor, MI, USA). The concentration of the purified *Pg*MA was determined using the Bradford method [39] and analyzed with sodium dodecyl sulfate (SDS) polyacrylamide gel electrophoresis (PAGE). The purified *Pg*MA was stored in a final concentration of 50% glycerol at −80 °C before use.

### 2.6. Assay of Hydrolysis Activity

The standard reaction was performed with 1% (*w*/*v*) β-CD, 5.6 μg/mL *Pg*MA, and 50 mM of PB at pH 6 and 60 °C for 30 min. After the reaction was stopped by boiling, the amount of reducing sugars produced from each reaction was estimated using the dinitrosalicylic acid method [40]. One unit of MA activity was defined as the amount of the enzyme that released 1 μmol of reducing sugar as maltose per min under the assay condition described earlier. For optimal conditions, the reaction was further performed at different temperatures and pH values, including pH 5 (acetate buffer), pH 6–7 (PB), and pH 8–10 (glycine buffer). Accordingly, the substrate specificity was measured with 1% (*w*/*v*) of the studied sugars, including α-CD, β-CD, γ-CD, soluble starch, and pullulan, performed at pH 7 and 65 °C. To realize the effects of metal ions and DMSO on the hydrolysis activity of *Pg*MA, 10 mM of tested metal ion or 5–20% (*v*/*v*) of DMSO was added into the reaction mixture.

### 2.7. Assay of Transglycosylation Activity

To determine the transglycosylation activity of the *Pg*MA, β-CD was used as a sugar donor, and the molecules, which belonged to triterpenoids, saponins, flavonoids, or polyphenol glycosides, were tested as sugar acceptors. The reaction mixture containing 5% (*w*/*v*) β-CD, 5.6 μg/mL *Pg*MA, and 1 mg/mL tested molecules, dissolved in DMSO with 50 mM of PB (pH 7), was incubated at 65 °C for 24 h. The reaction mixture was then mixed with an equal volume of methanol and analyzed with high-performance liquid chromatography (HPLC).

### 2.8. HPLC Analysis

HPLC was performed with the Agilent 1100 series HPLC system (Santa Clara, CA, USA) equipped with a gradient pump (Waters 600, Waters, Milford, MA, USA). The stationary phase was a C18 column (5 μm, 4.6 i.d. × 250 mm; Sharpsil H-C18, Sharpsil, Bei-jing, China), and the mobile phase was 1% acetic acid in water (A) and methanol (B). The elution condition was a linear gradient from 0 min with 40% B to 20 min with 70% B, an isocratic elution from 20 to 25 min with 70% B, a linear gradient from 25 min with 70% B to 28 min with 40% B, and an isocratic elution from 28 to 35 min with 40% B. All eluants were eluted at a flow rate of 1 mL/min. The sample volume was 10 μL. The detection condition was set at 254 nm.

### 2.9. Purification of Mangiferin Glycosides

The purification process was a previously described method [41]. A 100 mL reaction mixture containing 50% (*w*/*v*) maltodextrin, 1 mg/mL mangiferin, 5.6 μg/mL *Pg*MA, and 50 mM PB (pH 7) was incubated at 65 °C for 24 h. After the large-scale reaction, an equal volume of methanol was added to stop the transglycosylation. The mixture was then filtrated through a 0.2 μm nylon membrane, and the filtrate was injected in a preparative YoungLin HPLC system (YL9100, YL Instrument, Gyeonggi-do, South Korea) equipped with a preparative C18 reversed-phase column (10 μm, 20.0 i.d. × 250 mm, ODS 3; Inertsil, GL Sciences, Eindhoven, The Netherlands) for the purification of biotransformation products. The operational conditions for the preparative HPLC analysis were the same as those in the HPLC analysis. The elution corresponding to the peak of the metabolite in the HPLC analysis was collected, condensed under a vacuum, and then crystallized by freeze drying. Finally, 20.1 mg of compound (**1**) and 9.3 mg of compound (**2**) were obtained, and the structures of the compounds were confirmed with nucleic magnetic resonance (NMR) and mass spectral analyses. The mass analysis was performed using the Finnigan LCQ Duo mass spectrometer (ThermoQuest Corp., San Jose, CA, USA) with electrospray ionization (ESI). ^1^H- and ^13^C-NMR, distortionless enhancement by polarization transfer (DEPT), heteronuclear single quantum coherence (HSQC), heteronuclear multiple bond connectivity (HMBC), correlation spectroscopy (COSY), and nuclear Overhauser effect spectroscopy (NOESY) spectra were recorded on a Bruker AV-700 NMR spectrometer at ambient temperature. Standard pulse sequences and parameters were used for the NMR experiments, and all chemical shifts were reported in parts per million (ppm, *δ*).

The composition of compound (**1**) was as follows: light yellow powder; mp 233-235 °C; ESI/MS *m/z*: 583.4 [M-H]^−^, 565.3, 331.0, 300.9, 259.3; ^1^H-NMR (DMSO-*d_6_*, 700 MHz): H*δ* 3.05 (1H, t, *J* = 5.6 Hz, H-4″), 3.15 (1H, d, *J* = 6.3 Hz, H-2″), 3.20 (1H, t, *J* = 9.1 Hz, H-3′), 3.29 (1H, t, *J* = 9.1 Hz, H-4′), 3.33 (1H, m, H-5′), 3.35 (1H, m, H-5″), 3.38 (1H, m, H-3″), 3.46 (1H, m, H-6″a), 3.52 (1H, d, *J* = 9.1 Hz, H-6″b), 3.62 (1H, d, *J* = 9.8 Hz, H-6′a), 3.70 (1H, dd, *J* = 11.2, 4.2 Hz, H-6′b), 4.02 (1H, br, H-2′), 4.58 (1H, d, *J* = 9.1 Hz, H-1′), 4.73 (1H, *J* = 4.2 Hz, H-1″), 6.36 (1H, s, H-4), 6.86 (1H, s, H-5), and 7.37 (1H, s, H-8). ^13^C-NMR (DMSO-*d_6_*, 175 MHz): C*δ* 60.6 (C-6″), 66.9 (C-6′), 70.0 (C-4″), 70.2 (C-2′, 4′), 72.1 (C-2″), 72.5 (C-3″), 73.2 (C-1′), 73.3 (C-5″), 78.9 (C-3′), 79.7 (C-5′), 93.3 (C-4), 98.7 (C-1″), 101.3 (C-9a), 102.6 (C-5), 107.5 (C-2), 108.1 (C-8), 111.8 (C-8a), 143.7 (C-7), 150.8 (C-10a), 154.0 (C-6), 156.2 (C-4a), 161.7 (C-1), 163.8 (C-3), and 179.1 (C-9).

The composition of compound (**2**) was as follows: light yellow powder; mp 227-229 °C; ESI/MS *m/z*: 745.3 [M-H]^−^, 727.3, 403.3, 385.0, 331.0, 313.2, 301.2; ^1^H-NMR (DMSO-*d_6_*, 700 MHz): H*δ* 3.03 (1H, t, *J* = 9.1 Hz, H-4‴), 3.19 (1H, dd, *J* = 9.1, 3.5 Hz, H-2‴), 3.21 (1H, m, H-3′), 3.23 (1H, m, H-2″), 3.30 (1H, m, H-4′), 3.34 (1H, m, H-4″), 3.36 (1H, m, H-5′), 3.38 (1H, m, H-3‴), 3.41 (1H, br, H-2′), 3.42 (1H, m, H-5‴), 3.44 (2H, m, H-6‴), 3.46 (1H, m, H-5″), 3.55 (2H, m, H-6″), 3.60 (1H, m, H-3″), 3.64 (1H, m, H-6′a), 3.71 (1H, m, H-6′b), 4.58 (1H, d, *J* = 9.8 Hz, H-1′), 4.75 (1H, d, *J* = 3.5 Hz, H-1″), 4.95 (1H, d, *J* = 3.5 Hz, H-1‴), 6.36 (1H, s, H-4), 6.86 (1H, s, H-5), and 7.37 (1H, s, H-8). ^13^C-NMR (DMSO-*d_6_*, 175 MHz): C*δ* 60.0 (C-6″), 60.7 (C-6‴), 67.2 (C-6′), 69.8 (C-4‴), 70.2 (C-2′, 4′), 70.8 (C-5″), 71.6 (C-2″), 72.6 (C-2‴), 73.1 (C-3″), 73.2 (C-1′), 73.3 (C-3‴), 73.4 (C-5‴), 78.9 (C-3′), 79.7 (C-5′), 79.8 (C-4″), 93.3 (C-4), 98.6 (C-1″), 100.9 (C-1‴), 101.3 (C-9a), 102.6 (C-5), 107.4 (C-2), 108.1 (C-8), 111.7 (C-8a), 143.7 (C-7), 150.8 (C-10a), 154.0 (C-6), 156.2 (C-4a), 161.8 (C-1), 163.8 (C-3), and 179.1 (C-9).

### 2.10. Determination of Aqueous Solubility

The aqueous solubility of mangiferin and its glucoside derivative were examined as follows: each compound was vortexed in double-deionized H_2_O for 1 h at 25° C. The mixture was centrifuged at 10,000× *g* for 30 min at 25 °C. The supernatant was filtrated with 0.2 μM of nylon membrane and analyzed with HPLC. Based on their peak areas, the concentrations of the tested compounds were determined by using calibration curves prepared with HPLC analyses of authentic samples. 

### 2.11. Determination of Antiradical Activity Using a DPPH Assay

The assay was performed as previously described [42] with minor modifications. The tested sample (dissolved in DMSO) was added to the DPPH solution (1 mM in methanol) to a final volume of 0.1 mL. After 30 min of reaction, the absorbance of the reaction mixture was measured at 517 nm with a microplate reader (Sunrise, Tecan, Männedorf, Switzerland). Ascorbic acid (dissolved in DMSO) was used as a positive antioxidant standard. The DPPH free radical scavenging activity was calculated as follows: DPPH free radical scavenging activity = (OD_517_ of the control reaction − OD_517_ of the reaction)/(OD_517_ of the control reaction).

## 3. Results and Discussion

### 3.1. Selection of Candidate MAs via Online Genome Sequences

**Figure 1:**
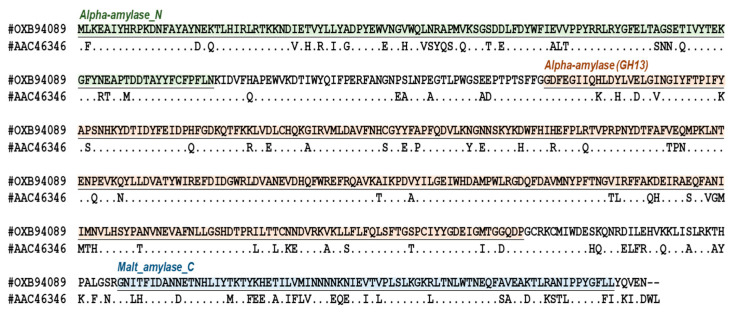
*Pg*MA (OXB94089) protein sequence. The GH13 family gene sequence identity between *Pg*MA and BsMT (AAC46346) is 79.08% (465/588). The Pfam domains are as follows: (1) alpha-amylase_N (alpha amylase, N-terminal IG-like domain; pfam02903): 1–121 amino acids; (2) alpha-amylase (alpha amylase catalytic domain found in cyclomaltodextrinases and related proteins; cd11338): 173–469 amino acids; and (3) malt_amylase_C (maltogenic amylase, C-terminal domain; pfam16657), 507–583 amino acids. “.” Denotes an identical amino acid; “-” denotes insertions and deletions.

Maltogenic amylase from *Bacillus stearothermophilus* (*Bs*MA, GenBank accession number: AAC46346) is a well-studied MA for the glycosylation of bioactive molecules [31,33,34,36,37]. Thus, the amino acid sequence of *Bs*MA was selected to search for new MAs from the NCBI GenBank. The highest but distinct MA sequences from bacterial whole genomes were further identified as our study’s candidates. The available bacterial strains with known genome sequences in BCRC (Hsinchu, Taiwan) were also included for comparison. Accordingly, an α-glycosidase gene (GenBank accession number: OXB94089.1) from the genome data of *P. galactosidasius* DSM 18751T (GenBank accession number: PRJNA383662) showed the highest homology (79.1%) with *Bs*MA (Figure 1) and the top five candidates with the best-hit of *Bs*MA from NCBI GenBank in Table S1. Therefore, the α-glycosidase gene from the DSM 18,751 strain was identified as a suitable candidate in the present study. Figure 1 shows the α-glycosidase gene from *P. galactosidasius* DSM 18751, which was classified as a maltogenic amylase gene, and the gene product was named *Pg*MA in this study.

### 3.2. Production of Recombinant PgMA in E. coli

**Figure 2:**
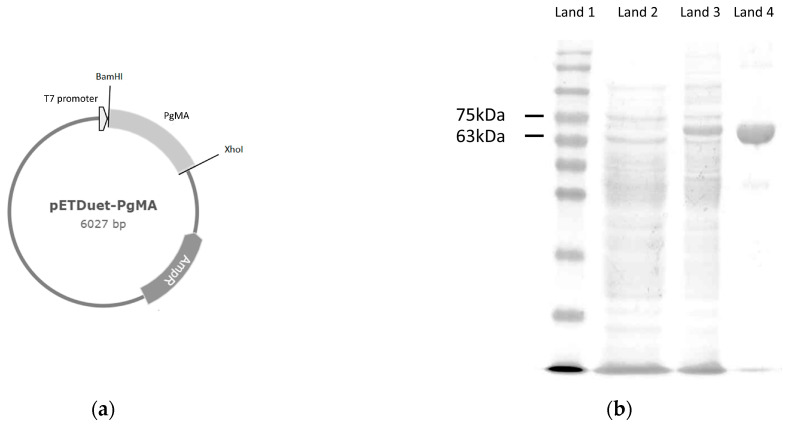
Expression of *Pg*MA from *Parageobacillus galactosidasius* DSM 18751T in *Escherichia coli* (DE3). (**a**) Recombinant repression plasmid pETDuet-*Pg*MA. (**b**) Sodium dodecyl sulfate-polyacrylamide gel electrophoresis (SDS-PAGE) analysis of expressed and purified proteins from recombinant *E. coli* harboring pETDuet-*Pg*MA. Lane 1: molecular marker; lane 2: total protein before induction; lane 3: total protein after 20 h of induction; and lane 4: purified protein.

To produce the recombinant *Pg*MA, an expression plasmid pETDuet-*Pg*MA was constructed (Figure 2a), and the recombinant *Pg*MA was produced in recombinant *E. coli* BL21 (DE3) and purified as a major band shown by SDS-PAGE with a Ni^2+^ affinity chromatography (Figure 2b). The purified *Pg*MA showed an estimated 68 kD molecular weight in the SDS-PAGE. The production yield was 18.73 mg/L, and the specific activity of the purified enzyme was determined to be 91.46 U/mg by using β-CD as a substrate at pH 7 and 60 °C.

### 3.3. Determination of Hydrolysis Activity by Recombinant PgMA

**Figure 3:**
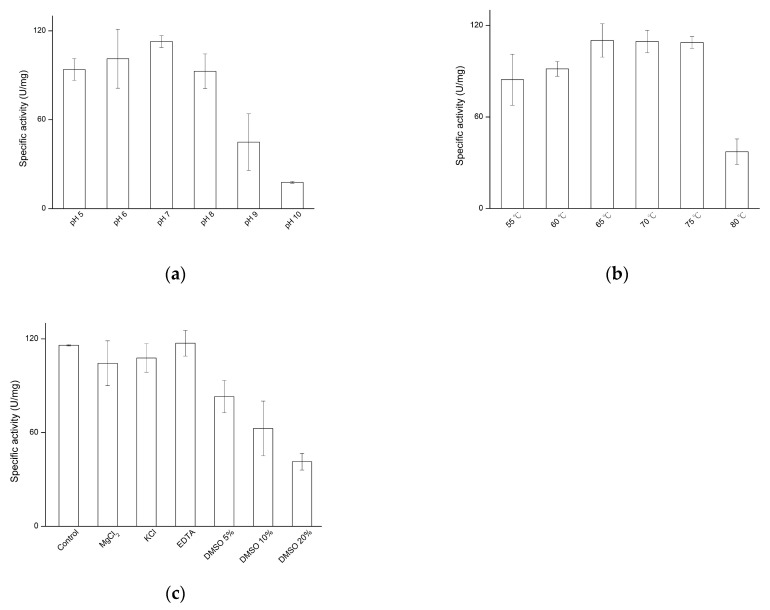
Effects of pH (**a**), temperature (**b**), and metal ion or dimethyl sulfoxide (DMSO) (**c**) on the hydrolytic activity of *Pg*MA. The reaction was performed with 1% (*w*/*v*) β-cyclodextrin (CD), 5.6 μg/mL of *Pg*MA, and 50 mM of a different buffer at the tested temperature in the absence or presence of the tested metal ion or DMSO for 30 min. After the reaction was stopped by boiling, the hydrolytic activity of *Pg*MA was determined by measuring the reducing sugars produced from the reaction as described in Section 2.

To determine the optimal pH and temperature, β-CD was used as a substrate, and the reaction was performed at different pH levels and temperatures. The results showed that the optimal pH and temperature for the *Pg*MA catalytic reaction were pH 7 (Figure 3a) at 65 °C (Figure 3b). Moreover, the addition of Mg^2+^, K^+^, or ethylenediaminetetraacetic acid did not significantly affect the activity of *Pg*MA; by contrast, DMSO decreased the activity of *Pg*MA (Figure 3c).

**Figure 4:**
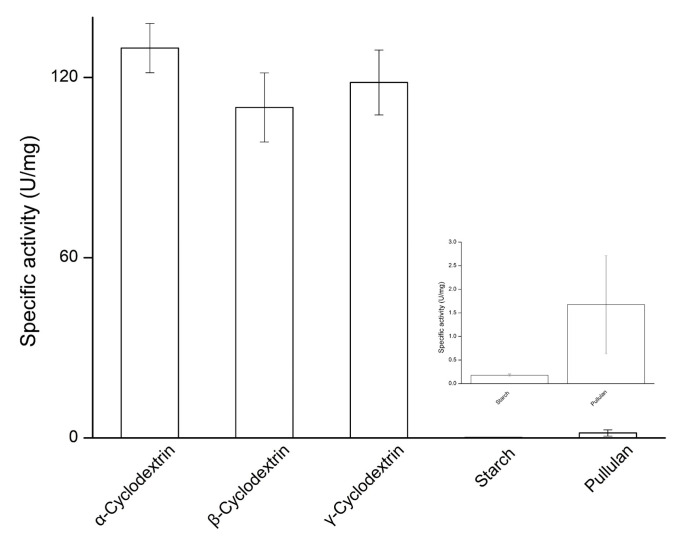
Substrate specificity of the hydrolytic activity of *Pg*MA. The reaction was performed with 1% (*w*/*v*) of the tested sugar substrate, 5.6 μg/mL *Pg*MA, and 50 mM of PB at pH 7 and 65 °C for 30 min. The hydrolytic activity of *Pg*MA was determined as described in the legend of Figure 3.

One of the specific features of MA is that the enzyme prefers CD as its substrate over other polysaccharides, such as starch or pullulan. To characterize the *Pg*MA hydrolysis activity, different polysaccharides were used as a substrate for the *Pg*MA catalytic reaction. The results showed that *Pg*MA exhibited almost equally high specific activities toward α-CD, γ-CD, and β-CD. The specific activities of *Pg*MA toward CD were 65- and 650-fold higher than those toward pullulan and starch, respectively (Figure 4). This CD preference was consistent with other known MAs [20,21,22,23,24,25,26,27]. The 130 residues at the N-terminal of the MA are key for the enzymes to form dimers and largely increase hydrolysis activities toward CD [18,19]. In addition, some recombinant MAs have been purified using N-terminal His-tag fusion [18,19,20,21,22,23,24,25,26,27]. The N-terminal His-tag fusion did not seem to affect its dimerization; herein, the recombinant *Pg*MA also remained as the CD preference.

### 3.4. Determination of Transglycosylation Activity by Recombinant PgMA

**Figure 5:**
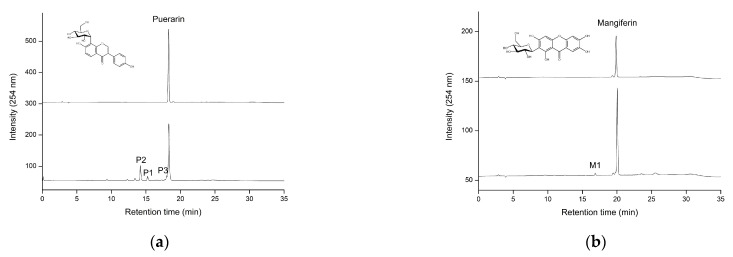
High-performance liquid chromatography (HPLC) analysis of the biotransformation products of puerarin (**a**) and mangiferin (**b**) by *Pg*MA. The reaction was performed with 1% (*w*/*v*) β-CD, 5.6 μg/mL of *Pg*MA, and 1 mg/mL of puerarin or mangiferin at 50 mM of PB (pH 7) and 65 °C for 24 h. After the reaction, the reaction mixture was analyzed with HPLC. The conditions for HPLC are described in Section 2.

Transglycosylation activity is an important property of MAs for biotechnology applications. To clarify the transglycosylation activity of recombinant *Pg*MA, 17 different molecules, including mangiferin (Table S2), belonging to triterpenoids, saponins, flavonoids, flavonoid glycosides, or xanthone glycoside, were used as sugar acceptors with 1% (*w*/*v*) β-CD (as the sugar donor) for activity. The reaction mixture was then analyzed with HPLC. The results showed that only puerarin (Figure 5a) and mangiferin (Figure 5b) could be glycosylated by *Pg*MA.

Except mangiferin and puerarin, the other four tested triterpenoids, two triterpenoids saponins, nine flavonoid aglycones, and glycosides could not act as the sugar acceptors in the transglycosylation of *Pg*MA. *Pg*MA could transglycosylate puerarin, which has the isoflavone-8-*C*-glucosdie structure. However, *Pg*MA could not transglycosylate isoflavone-7-*O*-glucoside (8-hydroxydaidzein-7-*α*-*O*-glucoside) or flavone-8-*C*-glucoside (vitexin). The results imply that *Pg*MA has a narrow and/or specific substrate range. Nevertheless, the main finding is that *Pg*MA can glycosylate mangiferin, which will expand the biotechnological applications of MAs in the future. MAs have been proven to glycosylate some small molecules, such as hydroquinone [29], caffeic acid [30], ascorbic acid [31], puerarin [32,33,34], genistin [35], neohesperidin [36], and naringin [37]. Our results also showed that *Pg*MA glycosylated puerarin to three major products, P1, P2, and P3 (Figure 5a). These three major products were not identified in advance because the glycosylation of puerarin has been studied based only on known MAs [32,34]. Li et al. (2004) reported that *Bs*MA glycosylated puerarin to three products (T1, T2, and T3), two of which were identified as maltosyl-α-(1→6)-puerarin (T1) and glucosyl-α-(1→6)-puerarin (T2), while T3 was not identified [34]. Li et al. (2011) further reported that a maltogenic amylase (*Tf*MA) from the archaeon *T. pendens* glycosylated puerarin to a series of products containing glucosyl puerarin and maltosyl puerarin, although they did not identify the exact chemical structures of the products [32]. From the results of the two studies, the P1–P3 products might contain glucosyl and maltosyl puerarin.

The results revealed that *Pg*MA glycosylates mangiferin to produce low amounts of the M1 compound with a yield of 2.3% (Figure 5b). This is the first study to report that MA could glycosylate mangiferin, of which mangiferin glycoside may have better aqueous solubility for different applications. Therefore, we mainly focused on the unknown mangiferin glycoside by *Pg*MA in the following assays.

### 3.5. Optimization of Biotransformation of Mangiferin by PgMA

**Figure 6:**
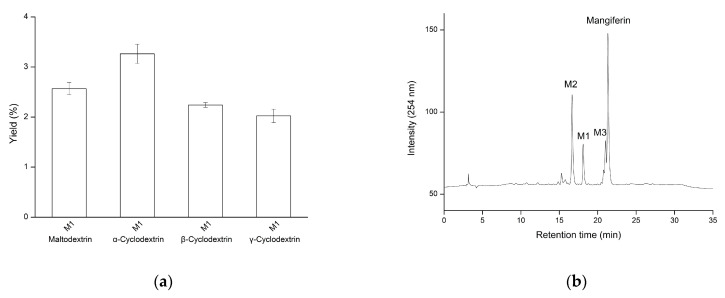

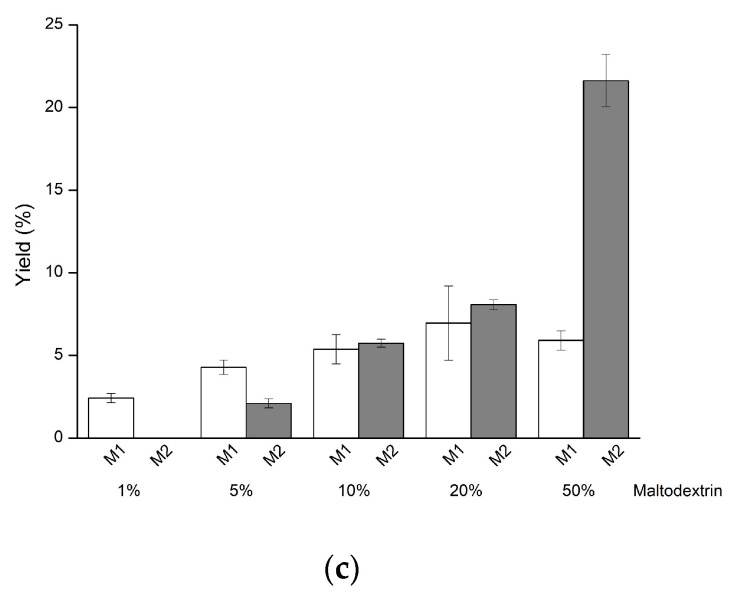
Effects of the sugar donor on the glycosylation of mangiferin by *Pg*MA. (**a**) Different sugar donors were used in the glycosylation of mangiferin. (**b**) HPLC analysis of the glycosylation mixture of mangiferin by *Pg*MA with 50% (*w*/*v*) maltodextrin. (**c**) Different maltodextrin concentrations were used in the glycosylation of mangiferin. The reaction was performed with 5.6 μg/mL *Pg*MA, 1 mg/mL mangiferin, and the tested sugar donors at 50 mM of PB (pH 7) and 65 °C for 24 h. After the reaction, the reaction mixture was analyzed with HPLC. The conditions for HPLC are described in Section 2. The yield of the product was calculated by dividing the area of the product by that of mangiferin without an enzyme reaction (control reaction).

As the yield of the M1 compound from the biotransformation by *Pg*MA is too low to be easily purified, the glycosylation condition must be optimized. The glycosylation reaction was optimized with different sugar donors, concentrations of sugar donors, and reaction times. First, although the yield of the M1 using α-CD showed the best output (Figure 6a), maltodextrin was selected as the sugar donor for experiments due to its highly aqueous solubility at a much lower price.

Second, the GH enzymes contained both hydrolysis and transglycosylation activities. The transglycosylation activity of GH has been reported to increase under low water concentrations [43]. Thus, a solution with a higher sugar concentration and lower water concentration would increase its transglycosylation activity. The transglycosylation activity of *Pg*MA was indeed increased in 50% maltodextrin, the highest soluble concentration. When the maltodextrin concentration was increased from 1% to 50% (*w*/*v*), three compounds (M1, M2, and M3 in Figure 6b) were formed with higher yields (Figure 6c). The maximal yields of M1 and M2 reached 10% and 21%, respectively. The M3 compound was not completely separated with mangiferin, which is similar to the situation of the T3 compound with puerarin by *Bs*MA [34]. Therefore, only M1 and M2 were further studied.

**Figure 7:**
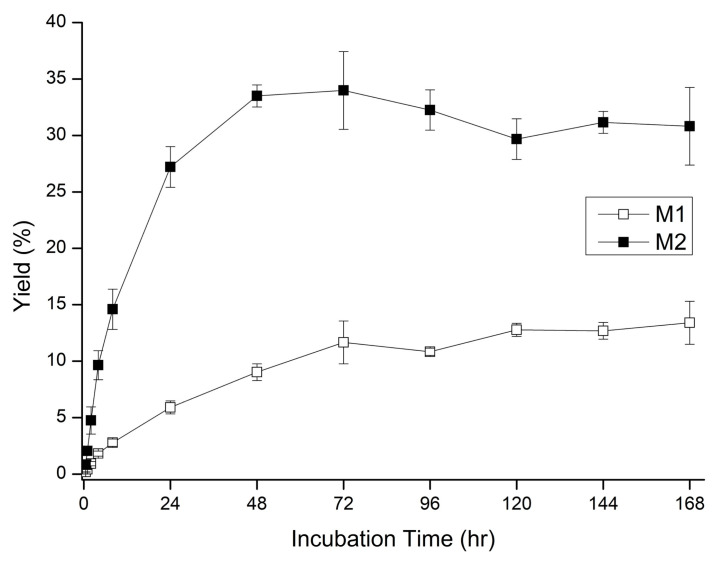
Time course of the glycosylation of mangiferin by *Pg*MA. The reaction was conducted with 50% (*w*/*v*) maltodextrin, 5.6 μg/mL *Pg*MA, and 1 mg/mL mangiferin at 50 mM of PB (pH 7) and 65 °C. At the interval time, the reaction mixture was analyzed with HPLC. The conditions for HPLC and the calculation of the yield were the same as those described in the legend to Figure 6.

Third, the yields of M1 and M2 in 50% maltodextrin by *Pg*MA were further determined under different time courses (Figure 7). The results showed that the yields plateau of M1 and M2 reached 13.2% at 168 h and 33.8% at 72 h, respectively.

### 3.6. Isolation and Identification of Mangiferin Glycosides by PgMA

The glycosylation of mangiferin by *Pg*MA was scaled up to 100 mL. The products M1 and M2 were purified using preparative HPLC. From the 100 mL reaction, 20.1 mg of compound (**1**) (M1) and 9.3 mg of compound (**2**) (M2) were isolated. The molecular weights of the purified products were then determined with mass spectrometry. The mass spectrometry of compound (**1**) revealed an [M–H]^−^ ion peak at *m/z*: 583.4 in the electrospray ionization mass spectrum (ESI-MS) corresponding to the molecular formula C_25_H_28_O_16_ (Figure S1). The mass data imply that M1 contains one glucosyl moiety attached to the mangiferin structure. In the mass data of M2, an [M–H]^−^ ion peak at *m/z*: 745.3 in the ESI-MS corresponded to the molecular formula C_31_H_38_O_21_ (Figure S2), which implies that compound (**2**) contains two glucosyl moieties attached to the mangiferin structure. To identify the structures in advance, the structures of both compounds were determined using NMR spectroscopy. ^1^H and ^13^C NMR, including the DEPT, HSQC, HMBC, COSY, and NOESY spectra, were obtained.

The characteristic ^1^H and ^13^C NMR sugar signals in compound (**1**) were assigned to *C*-glucosyl and *O*-glucosyl moieties by one-dimensional (1-D) and 2-D NMR experiments. The ^1^H spectrum of compound (**1**) in DMSO-*d_6_* showed three singlets at 6.36, 6.86, and 7.37 ppm and a complex 10-spin system between 3.0 and 5.0 ppm. Analysis of this second-order system revealed coupling constants typical of two glucose moieties. The compound (**1**) glucosidic linkage of the *C*-glucosyl moiety on the xanthone C-2 was revealed by the presence of HMBC correlations between C-2/H-1′ (107.5/4.58 ppm), and the anomeric proton H-1′ at 4.58 (d, *J* = 9.1 Hz) indicated a *C*-*β*- configuration of mangiferin that was confirmed by the data reported in the literature [44]. The mangiferin *O*-glucosyl moiety was a doublet signal at H-1″ (4.73 ppm, d, *J* = 4.2 Hz) with the corresponding carbon atom at C-1″ (98.7 ppm) assigned to the anomeric proton and indicating an *O*-*α*-configuration by HSQC, which is in the *O*-*α*-configuration. The H-1″ (*δ* = 4.73 ppm) of mangiferin and the HMBC cross signaled H-1″/C-6′ (4.73/66.9 ppm) and H-6′a, 6′b/C-1″ (3.46, 3.52/98.7 ppm). The significant downfield shift of the ^13^C signal of C-6′ indicated the connection of the second glucosyl moiety, which confirmed the *α*-(1→6) linkage of the second glucosyl moiety. The NMR signals were identified as shown in Table S3. The compound (1) was thus confirmed as glucosyl-*α*-(1→6)-mangiferin (Figures S3–S9).

**Figure 8:**
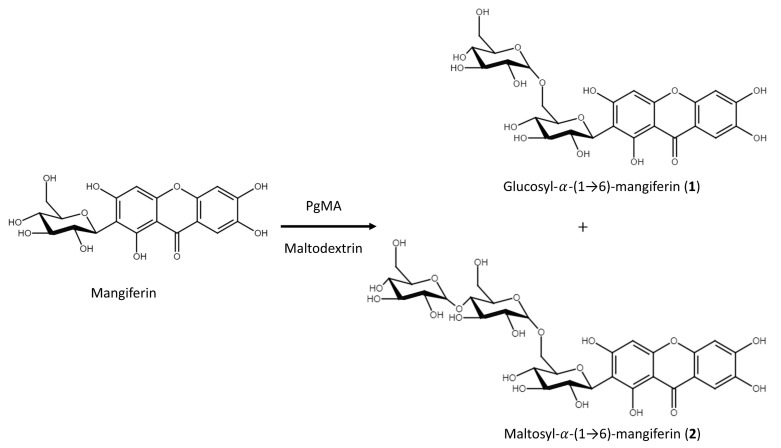
Biotransformation process of mangiferin by *Pg*MA.

The ^1^H spectrum of compound (**2**) in the same compound (**1**) solvent also showed three singlets at 6.36, 6.86, and 7.37 ppm and a complex 11-spin system between 3.0 and 5.0 ppm. Analysis of this second-order system revealed coupling constants typical of three glycose moieties, which included the chemical shifts listed in Table S3. The glucosyl moiety chemical shifts of C-2 at 107.4 ppm and H-1′ at 4.58 ppm (d, *J* = 9.8 Hz) according to the corresponding HMBC indicated a C-C bond between the sugar and the aglycone of mangiferin (*C*-glucosyl-xanthone) and were confirmed by the data reported in the literature [44]. The *O*-maltosyl moiety connected to mangiferin was confirmed by HMBC from the anomeric carbon C-6′ (66.9 ppm), and the corresponding anomeric proton H-1″ at 4.75 (d, *J* = 3.5 Hz) indicated an *O*-*α*-configuration. The maltose doublet signal at *δ*_H_ H-1″ (d, *J* = 3.5 Hz) and H-1‴ 4.95 (d, *J* = 3.5 Hz) with the corresponding carbon atom at C-1″ (98.6 ppm) and C-1‴ (100.9 ppm) was assigned to the anomeric proton and indicated two *O*-*α*-configurations by HSQC. The HMBC cross peaks of C-1″/H-6′ (98.6/3.64, 3.71 ppm) and C-1‴/H-4″ (100.9/3.34 ppm) confirmed the *α*-(1→4) between the two-glucosyl moiety. Our experimental ^1^H and ^13^C chemical shifts listed in Table S2 confirmed compound (**2**) as maltosyl-*α*-(1→6)-mangiferin (Figures S10–S16). Figure 8 summarizes the biotransformation process of mangiferin by *Pg*MA.

### 3.7. Characterizations of Mangiferin Glucosides

The low aqueous solubility of mangiferin restricts its usage as a pharmaceutical agent. However, the glycosylation of mangiferin may mitigate such a restriction. Only a few studies have reported different glycosylation agents for mangiferin. Wu et al. (2013) used β-fructofuranosidase (E.C. 3.2.1.26; GH 32 family) to glycosylate mangiferin into fructosyl-β-(2→6)-mangiferin and found that its DPPH radical scavenging activity was similar to that of mangiferin [45]. Nguyen et al. (2020) used dextransucrase (E.C. 2.4.1.5; GH 70 family) from *Leuconostoc mesenteroides* to glycosylate mangiferin into glucosyl-α-(1→6)-mangiferin (**1**) [46]. They found that the aqueous solubility of glucosyl-*α*-(1→6)-mangiferin (**1**) was 2300-fold higher than that of mangiferin. In this study, the amount of purified glucosyl-*α*-(1→6)-mangiferin (**1**) was too low to repeat the solubility experiment. The solubility of the newly identified maltosyl-*α*-(1→6)-mangiferin (**2**) was determined. The results showed that the solubility of maltosyl-*α*-(1→6)-mangiferin (**2**) was 5500-fold higher than that of mangiferin (Table 1). Thus, maltosyl-*α*-(1→6)-mangiferin (**2**) possesses higher solubility than glucosyl-*α*-(1→6)-mangiferin (**1**). It has been reported that the more sugar moieties in the glycosylated compounds, the higher the aqueous solubility of the glycosylated compounds [29,30,31,32,33,34,35,36,37].

**Figure 9:**
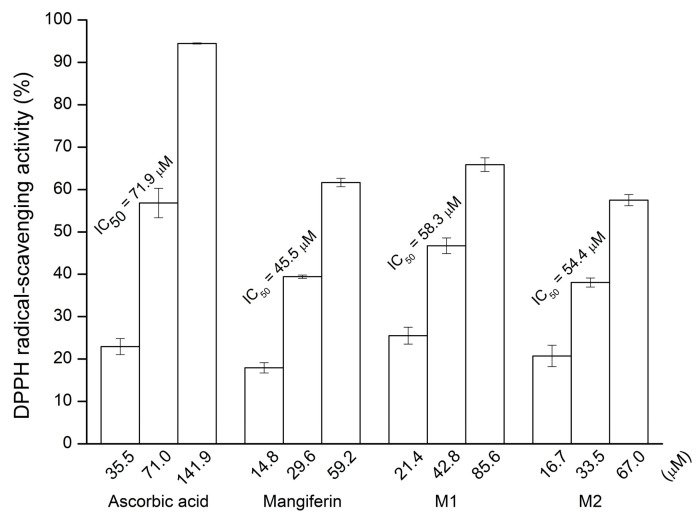
The 1,1-diphenyl-2-picrylhydrazyl (DPPH) free radical scavenging activity of mangiferin, mangiferin glucosides, and ascorbic acid. The DPPH scavenging activity was determined as described in Section 2. The IC_50_ values represent the concentrations required for 50% DPPH free radical scavenging activity. The mean (*n* = 3) is shown, and the standard deviations are represented by error bars. M1 and M2 are glucosyl-*α*-(1→6)-mangiferin and maltosyl-*α*-(1→6)-mangiferin, respectively.

Mangiferin exhibits a wide pharmacological profile, and its antioxidant property is well known from previous studies. Furthermore, it has been associated with the redox aromatic system of the xanthone nucleus [3,4,5,6,7,8,9,10,12]. Thus, the antioxidative activities of the two mangiferin glucosides were determined using DPPH free radical scavenging assay. The assay showed that the antioxidant activity levels of mangiferin and its two glucosides were all higher than those of ascorbic acid (Figure 9). In other words, the antioxidant activities of the two mangiferin glucosides are comparable with those of mangiferin. The *ortho*-dihydroxyl groups on the benzene ring of the mangiferin structure have been reported to play a key role in exerting its antioxidant activity [1,2]. Both mangiferin glucosides remained the key functional groups after glycosylation; therefore, most of the antioxidant activity remained in the mangiferin derivatives. These glycoside derivatives (glucoside and fructoside) might possess different pharmacological properties. A futher study will focus on the bioactivities and bioavailability of these mangiferin derivatives

## 4. Conclusions

The recombinant *Pg*MA from *P. galactosidasius* DSM 18751^T^ was confirmed to exhibit the bifunctions of hydrolysis and transglycosylation activities. The recombinant *Pg*MA can glycosylate mangiferin and produce glucosyl-*α*-(1→6)-mangiferin and maltosyl-*α*-(1→6)-mangiferin with a high maltodextrin concentration. The novel maltosyl-*α*-(1→6)-mangiferin showed much higher aqueous solubility than that of mangiferin. The two mangiferin glucosides exhibited similar DPPH antioxidative activity compared to mangiferin. To our knowledge, *Pg*MA is the first MA identified with glycosylation activity toward mangiferin. With higher water solubility and compatible antioxidant activity, the two mangiferin glucoside derivatives have better pharmaceutical applicability.

## Figures and Tables

**Table 1:** Aqueous solubility of mangiferin and maltosyl-*α*-(1→6)-mangiferin (**2**).

Compound	Aqueous Solubility (mg/L) ^1^	Fold ^2^
Mangiferin	92.2 ± 4.60	1.0
Maltosyl-*α*-(1→6)-mangiferin (2)	5.11 × 10^5^ ± 2.64 × 10^3^	5.5 × 10^3^

^1^ The mean (*n* = 2) is shown, and the standard deviations are represented by error bars. ^2^ The folds of the aqueous solubilities of the mangiferin glucoside derivatives are expressed as relative to that of mangiferin normalized to 1.

## Data Availability

The data presented in this study are available in the article or supplementary material.

## Supplementary Materials

The following materials are available online at https://www.mdpi.com/article/10.3390/antiox10111817/s1. Table S1: Top five candidates with best-hit of *Bs*MA from NCBI GenBank. Table S2. The list of the tested molecules in the transglycosylation reaction of *Pg*MA. Table S3: ^1^H and ^13^C NMR assignments in DMSO-*d_6_* at 700 and 175 MHz for compounds (**1**) and (**2**). Figure S1: Mass–mass analysis of glucosyl-*α*-(1→6)-mangiferin (**1**) in the negative mode. Figure S2: Mass–mass analysis of maltosyl-*α*-(1→6)-mangiferin (**2**) in the negative mode. Figure S3: One-dimensional NMR spectrum (^1^H-NMR, 700 MHz, DMSO-*d_6_*) of the glucosyl-*α*-(1→6)-mangiferin (**1**). Figure S4: One-dimensional NMR spectrum (^13^C-NMR, 175 MHz, DMSO- *d_6_*) of the glucosyl-*α*-(1→6)-mangiferin (**1**). Figure S5: One-dimensional NMR spectrum (DEPT-135, 175 MHz, DMSO-*d_6_*) of the glucosyl-*α*-(1→6)-mangiferin (**1**). Figure S6: Two-dimensional NMR spectrum (^1^H-^13^C HSQC, 700 MHz, DMSO-*d_6_*) of the glucosyl-*α*-(1→6)-mangiferin (**1**). Figure S7: Two-dimensional NMR spectrum (^1^H-^13^C HMBC, 700 MHz, DMSO-*d_6_*) of the glucosyl-*α*-(1→6)-mangiferin (**1**). Figure S8: Two-dimensional NMR spectrum (^1^H-^1^H COSY, 700 MHz, DMSO-*d_6_*) of the glucosyl-*α*-(1→6)-mangiferin (**1**). Figure S9: Two-dimensional NMR spectrum (^1^H-^1^H NOESY, 700 MHz, DMSO-*d_6_*) of the glucosyl-*α*-(1→6)-mangiferin (**1**). Figure S10: One-dimensional NMR spectrum (^1^H-NMR, 700 MHz, DMSO-*d_6_*) of the maltosyl-*α*-(1→6)-mangiferin (**2**). Figure S11: One-dimensional NMR spectrum (^13^C-NMR, 175 MHz, DMSO-*d_6_*) of the maltosyl-*α*-(1→6)-mangiferin (**2**). Figure S12: One-dimensional NMR spectrum (DEPT-135, 175 MHz, DMSO-*d_6_*) of the maltosyl-*α*-(1→6)-mangiferin (**2**). Figure S13: Two-dimensional NMR spectrum (^1^H-^13^C HSQC, 700 MHz, DMSO-*d_6_*) of the maltosyl-*α*-(1→6)-mangiferin (**2**). Figure S14: Two-dimensional NMR spectrum (^1^H-^13^C HMBC, 700 MHz, DMSO-*d_6_*) of the maltosyl-*α*-(1→6)-mangiferin (**2**). Figure S15: Two-dimensional NMR spectrum (^1^H-^1^H COSY, 700 MHz, DMSO-*d_6_*) of the maltosyl-*α*-(1→6)-mangiferin (**2**). Figure S16: Two-dimensional NMR spectrum (^1^H-^1^H NOESY, 700 MHz, DMSO-*d_6_*) of the maltosyl-*α*-(1→6)-mangiferin (**2**).
