# Supplementary material for: Improving Aqueous Solubility of Natural Antioxidant Mangiferin through Glycosylation by Maltogenic Amylase from Parageobacillus galactosidasius DSM 18751
Source: Antioxidants (Basel). 2021 Nov 16;10(11):1817. doi: 10.3390/antiox10111817 (PMC8615176; doi:10.3390/antiox10111817)
Supplement: Supplementary file 1 [file antioxidants-10-01817-s001.zip › antioxidants-1454036-supplementary.pdf]

Supplementary Materials: Improving Aqueous Solubility of Natural Antioxidant Mangiferin through Glycosylation by Maltogenic Amylase from *Parageobacillus galactosidasius* DSM 18751

Jiumn-Yih Wu, [1,+] Hsiou-Yu Ding, [2,+] Tzi-Yuan Wang, [3,+] Yu-Li Tsai, [4] Huei-Ju Ting [4] and Te-Sheng Chang [4,*]

[1] *Department of Food Science, National University of Quemoy, Kinmen, Taiwan*

[2] Department of Cosmetic Science, Chia Nan University of Pharmacy and Science, Tainan 717, Taiwan

[3] *Biodiversity Research Canter, Academia Sinica, Taipei 115, Taiwan*

[4] *Department of Biological Sciences and Technology, National University of Tainan, Tainan, Taiwan;*

[†] These authors contribute equally

[*] Correspondence: Te-Sheng Chang, mozyme2001@gmail.com

Table S1. Top five candidates with best-hit of *Bs*MA from NCBI GenBank.

**Resource Strain**	**Gene name**	**Gene accesses number**	**Identity to *Bs*MA**
Parageobacillus galactosidasius	α-Glycosidase	OXB94089.1	465/588 (79%)
Parageobacillus thermoglucosidasius	Cyclomaltodextrinase	ALF09762.1	450/589 (76%)
Anoxybacillus ayderensis	Neopullulanase	KIP21777.1	429/581 (74%)
Geobacillus stearothermophilus ATCC 12980	Cyclomaltodextrinase	KOR94460.1	415/589 (70%)
Bacillus cohnii	α-Glycosidase	AST90561.1	401/585 (69%)

Table S2. The list of the tested molecules in the transglycosylation reaction of *Pg*MA.

**Class**	**Name**	**Structure**
Triterpenoids	Antcin K	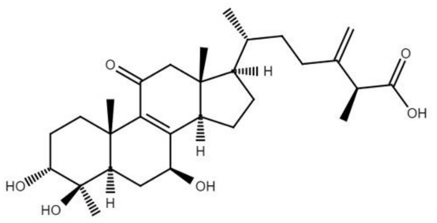
	Ganoderic acid A (GAA)	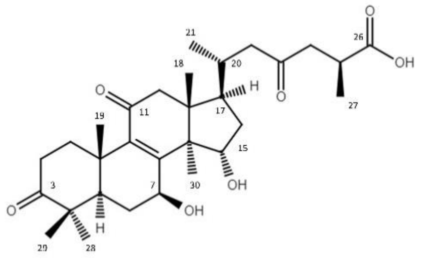
	Ganoderic acid G	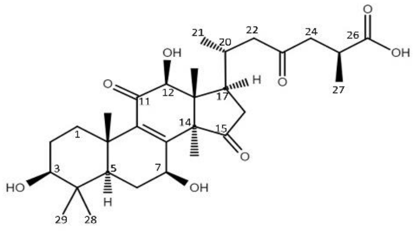
	Celastrol	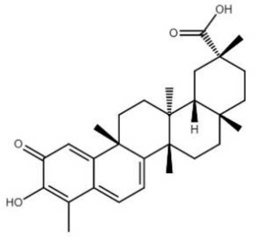
Triterpenoid Saponins	GAA-15-*O*-*β*-glucoside	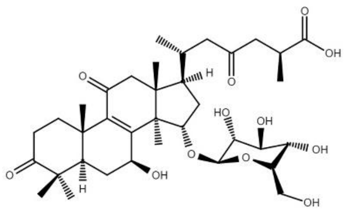

	GAA-15,26-*O*-*β*-diglucoside	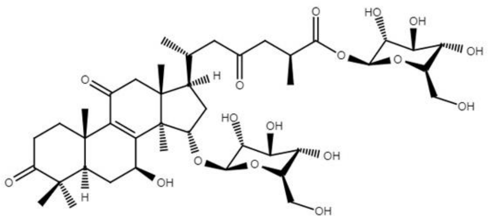
Flavonoids	Resveratrol	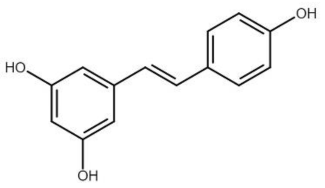
	Apigenin	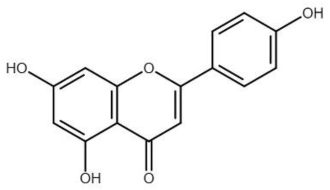
	Quercetin	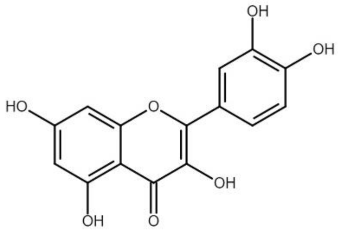
	Naringenin	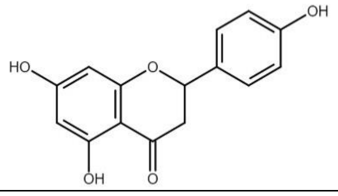
	Genistein	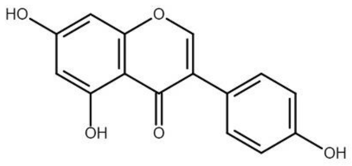
	Daidzein	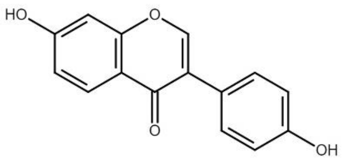
	8-Hydroxydaidzein (8-OHDe)	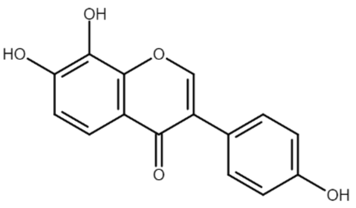

Flavonoid Glycosides	Naringin	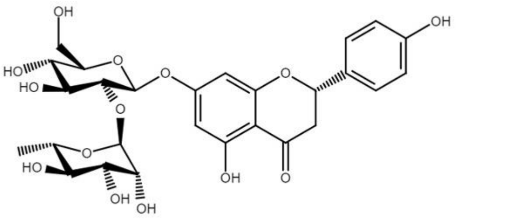
	8-OHDe-7-*O*-α-glucoside	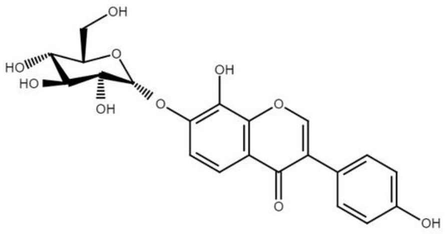
	Puerarin	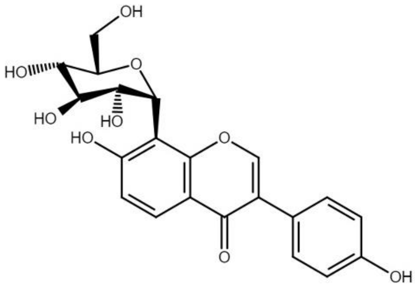
	Vitexin	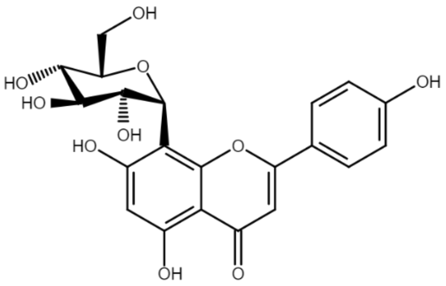
Xanthone Glycosides	Mangiferin	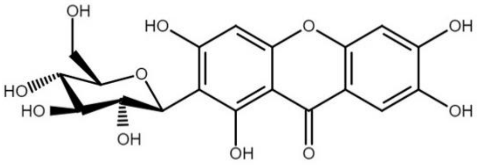

Table S3. ^{1}H and ^{13}C NMR assignments in DMSO-d_6 at 700 and 175 MHz for compounds (**1**) and (**2**). (δ in ppm, J in Hz).

position	compound **(1)**	compound (**2**)
1	161.7	161.8
2	107.5	107.4
3	163.8	163.8
4	93.3 (6.36, s)	93.3 (6.36, s)
4a	156.2	156.2
5	102.6 (6.86, s)	102.6 (6.86, s)
6	154.0	154.0
7	143.7	143.7
8	108.1 (7.37, s)	108.1 (7.37, s)
8a	111.8	111.7
9	179.1	179.1
9a	101.3	101.3
10	150.8	150.8
1'	73.2 (4.58, d, J=9.1 Hz)	73.2 (4.58, d, J=9.8 Hz)
2'	70.2 (4.02, br, s)	70.2 (3.41, br, s)
3'	78.9 (3.20, t, J=9.1 Hz)	78.9 (3.21, m)
4'	70.2 (3.29, t, J=9.1 Hz)	70.2 (3.30, m)
5'	79.7 (3.33, m)	79.7 (3.36, m)
6'	66.9 (3.62, d, J=9.1Hz; 3.70, dd, J=11.2, 4.2 Hz)	67.2 (3.64, m; 3.71, m)
1"	98.7 (4.73, d, J=4.2 Hz)	98.6 (4.75, d, J=3.5 Hz)
2"	72.1 (3.15, m)	71.6 (3.23, m)
3"	72.5 (3.38, m)	73.1 (3.60, m)
4"	70.0 (3.05, m)	79.8 (3.34, m)
5"	73.3 (3.35, m)	70.8 (3.46, m)
6"	60.6 (3.46, m; 3.52,m)	60.0 (3.55, m)
1"'		100.9 (4.95, d, J=3.5 Hz)
2"'		72.6 (3.19, dd, J=9.8, 3.5 Hz)
3"'		73.3 (3.38, m)
4"'		69.8 (3.03, t, J=9.1 Hz)
5"'		73.4 (3.42, m)
6"'		60.7 (3.44, m)

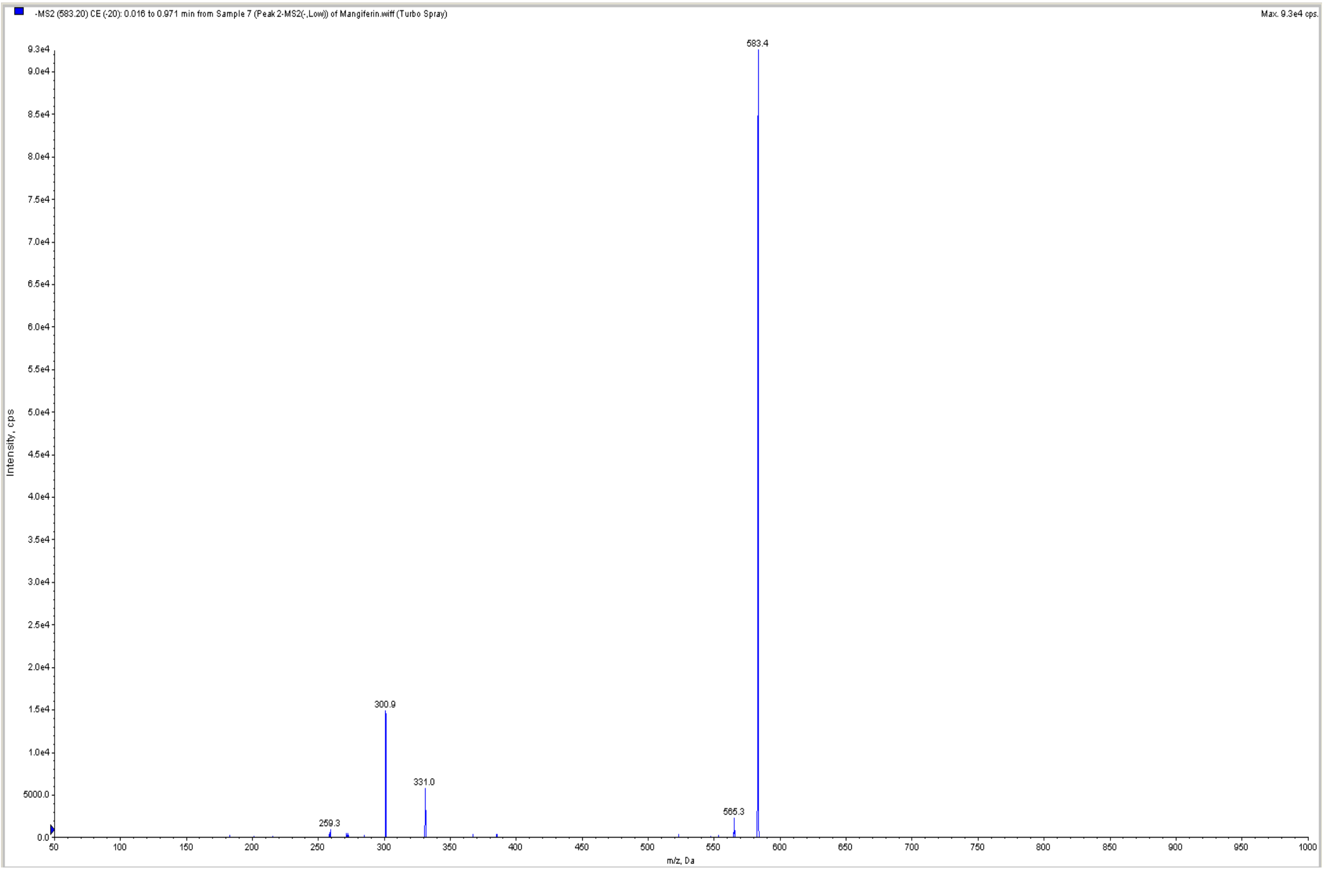

Figure S1. The mass-mass analysis of glucosyl-*α*-(1→6)-mangiferin (**1**) at the negative mode. A significant signal at *m*/*z* 583.4 showed the corresponding *m*/*z* signal of molecular weight 422 of glucosyl-*α*-(1→6)-mangiferin (**1**) (422+180-18-1) at the negative mode [mangiferin -G – H]$^-$.

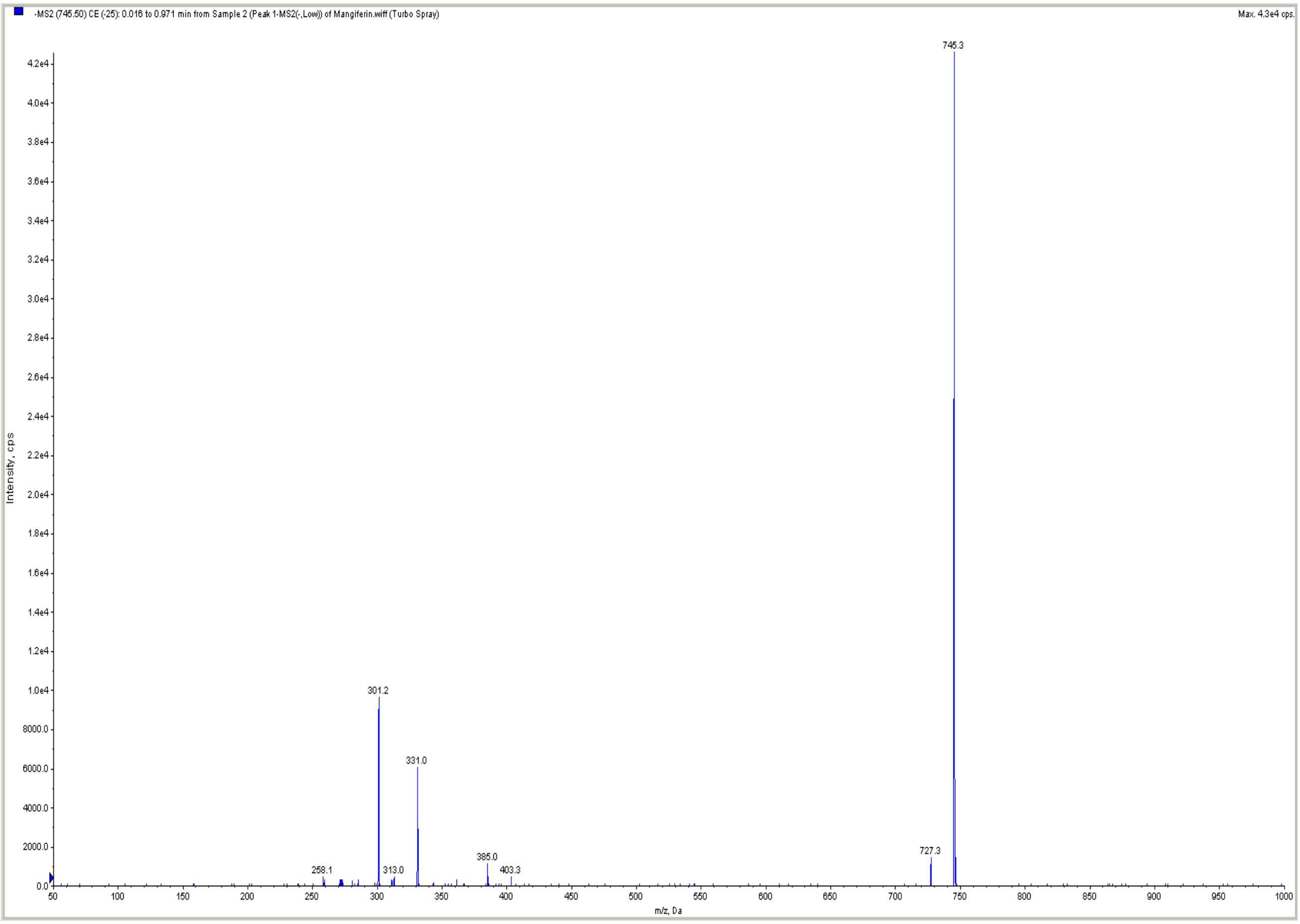

Figure S2. The mass-mass analysis of maltosyl-α-(1→6)-mangiferin (**2**) at the negative mode. A significant signal at *m*/*z* 745.3 showed the corresponding *m*/*z* signal of molecular weight 422 of maltosyl-α-(1→6)-mangiferin (**2**) (422+ 180 x 2 – 18 x 2 - 1 = 745) at the negative mode mangiferin -2G – H]$^-$.

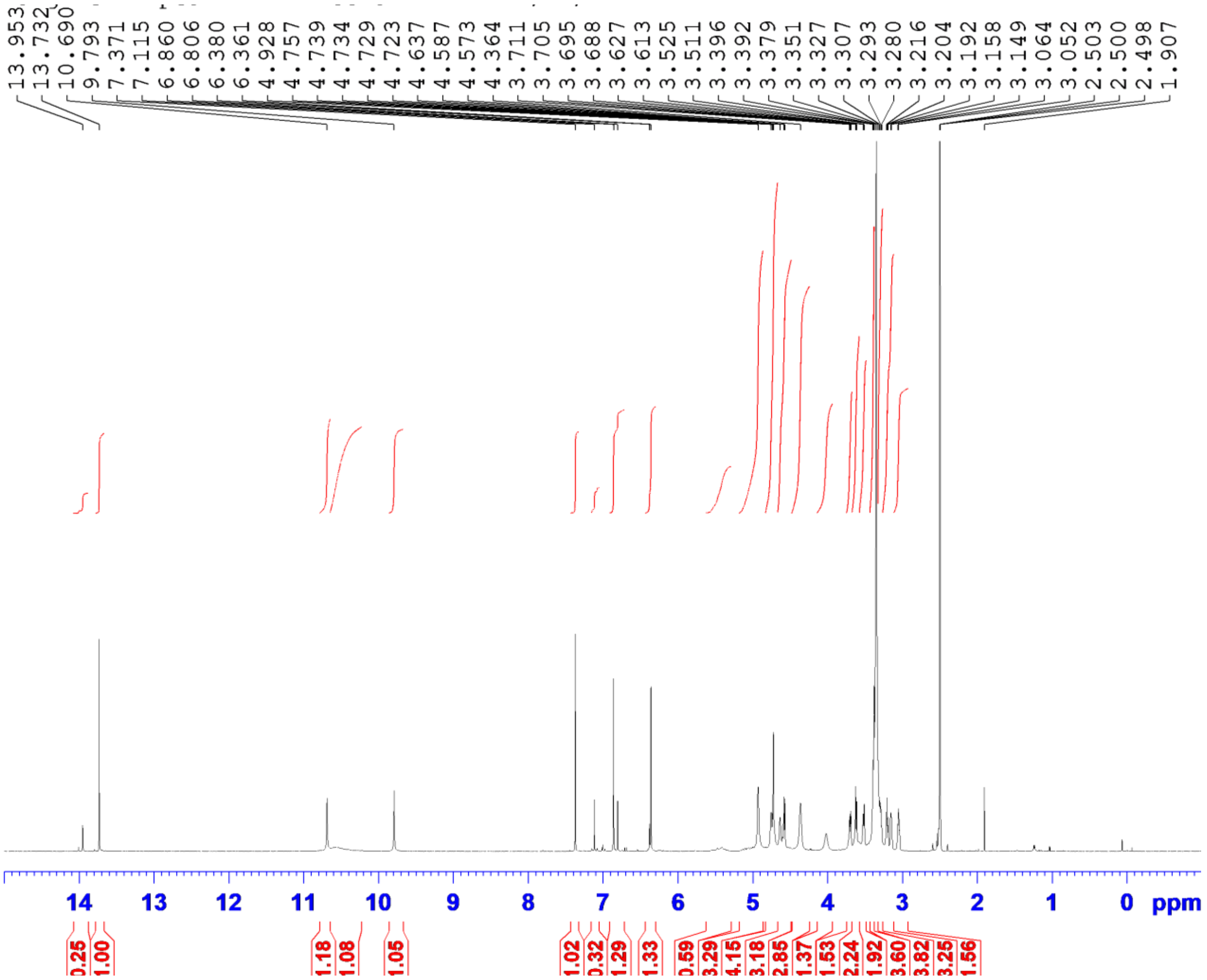

Figure S3. 1D NMR spectrum (^{1}H-NMR, 700 MHz, DMSO-d_6) of the glucosyl-α-(1→6)-mangiferin (**1**).

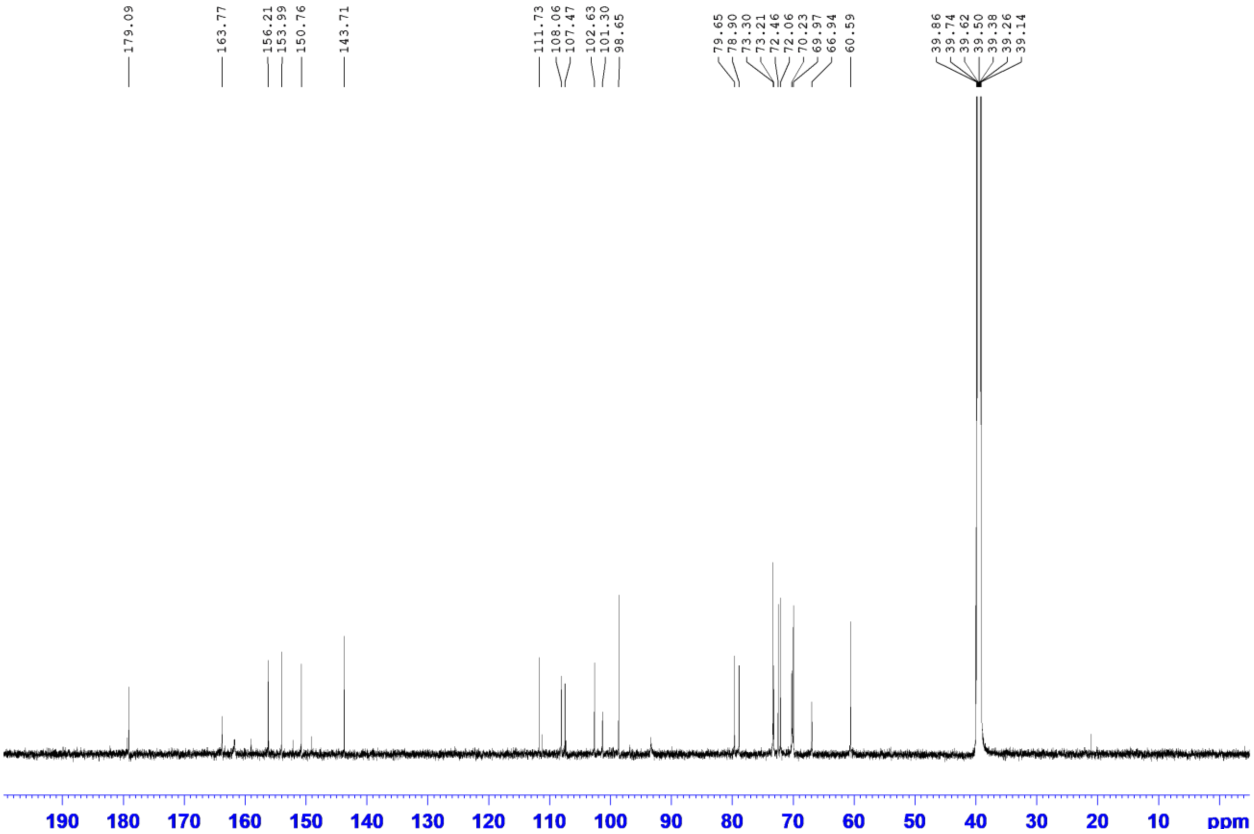

Figure S4. 1D NMR spectrum (^{13}C-NMR, 175 MHz, DMSO- d_6) of the glucosyl-α-(1→6)-mangiferin (**1**).

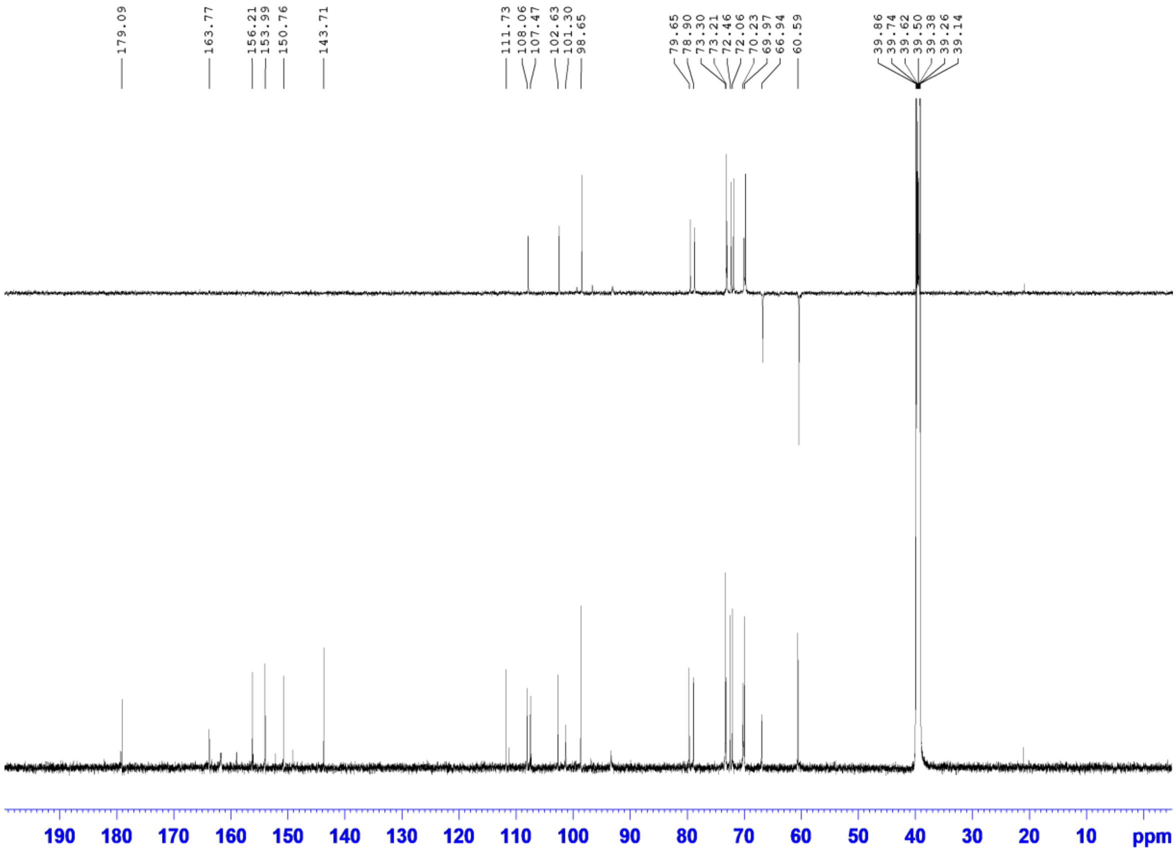

Figure S5. 1D NMR spectrum (DEPT-135, 175 MHz, DMSO-d_6) of the glucosyl-α-(1→6)-mangiferin (**1**).

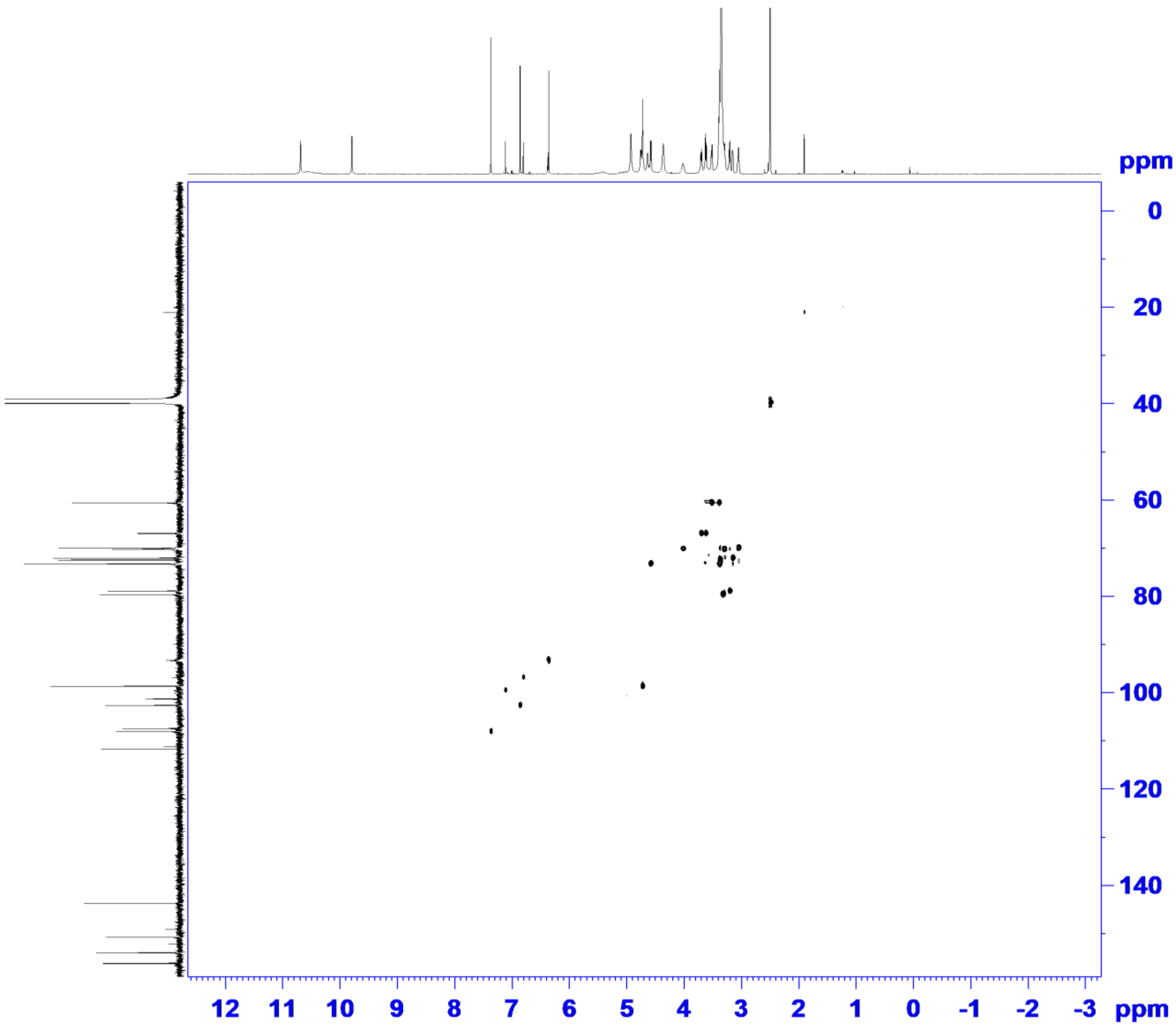

Figure S6. 2D NMR spectrum (^1H-^{13}C HSQC, 700 MHz, DMSO-d_6) of the glucosyl-α-(1→6)-mangiferin (**1**).

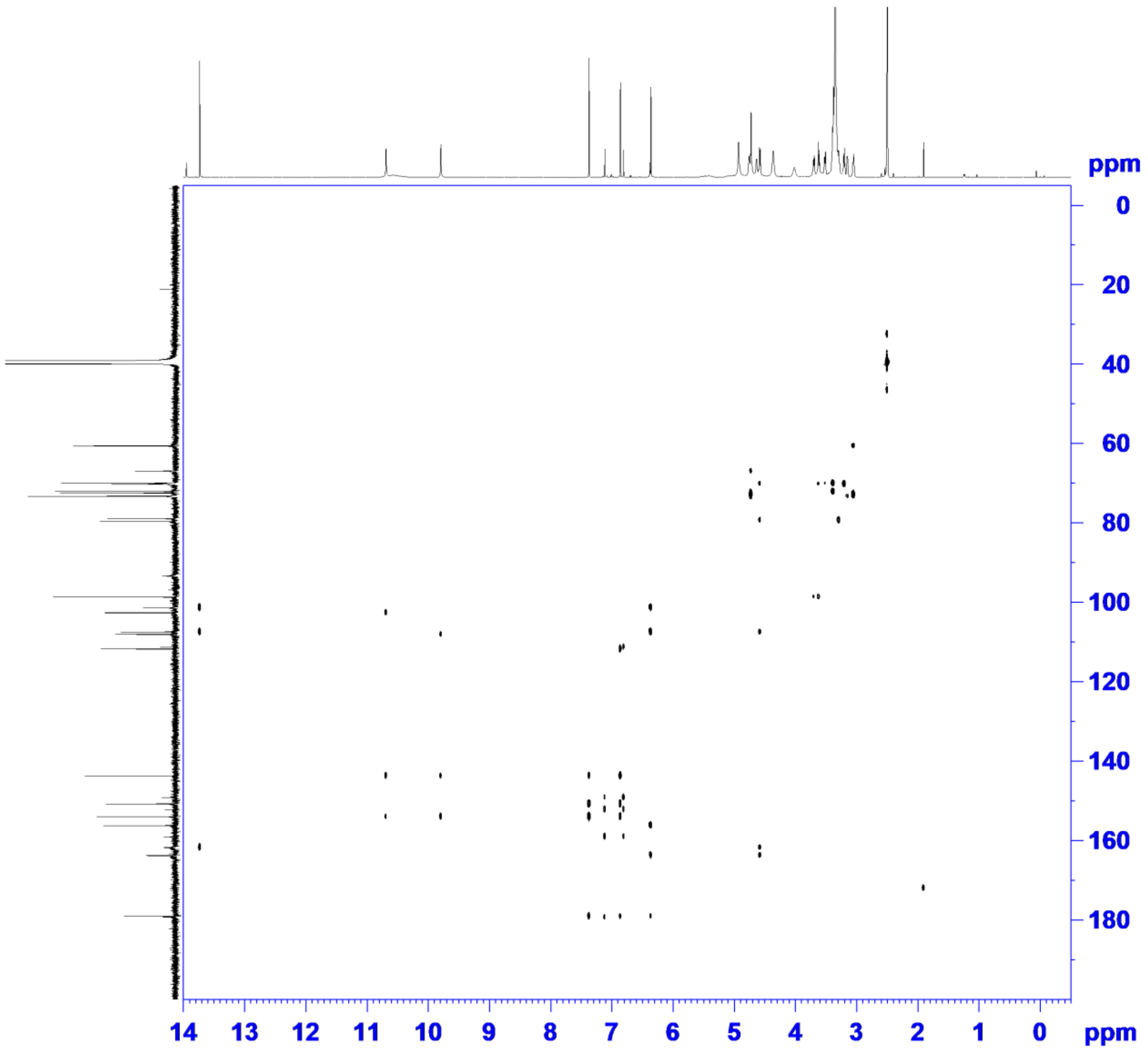

Figure S7. 2D NMR spectrum (^{1}H-^{13}C HMBC, 700 MHz, DMSO-d_6) of the glucosyl-α-(1→6)-mangiferin (**1**).

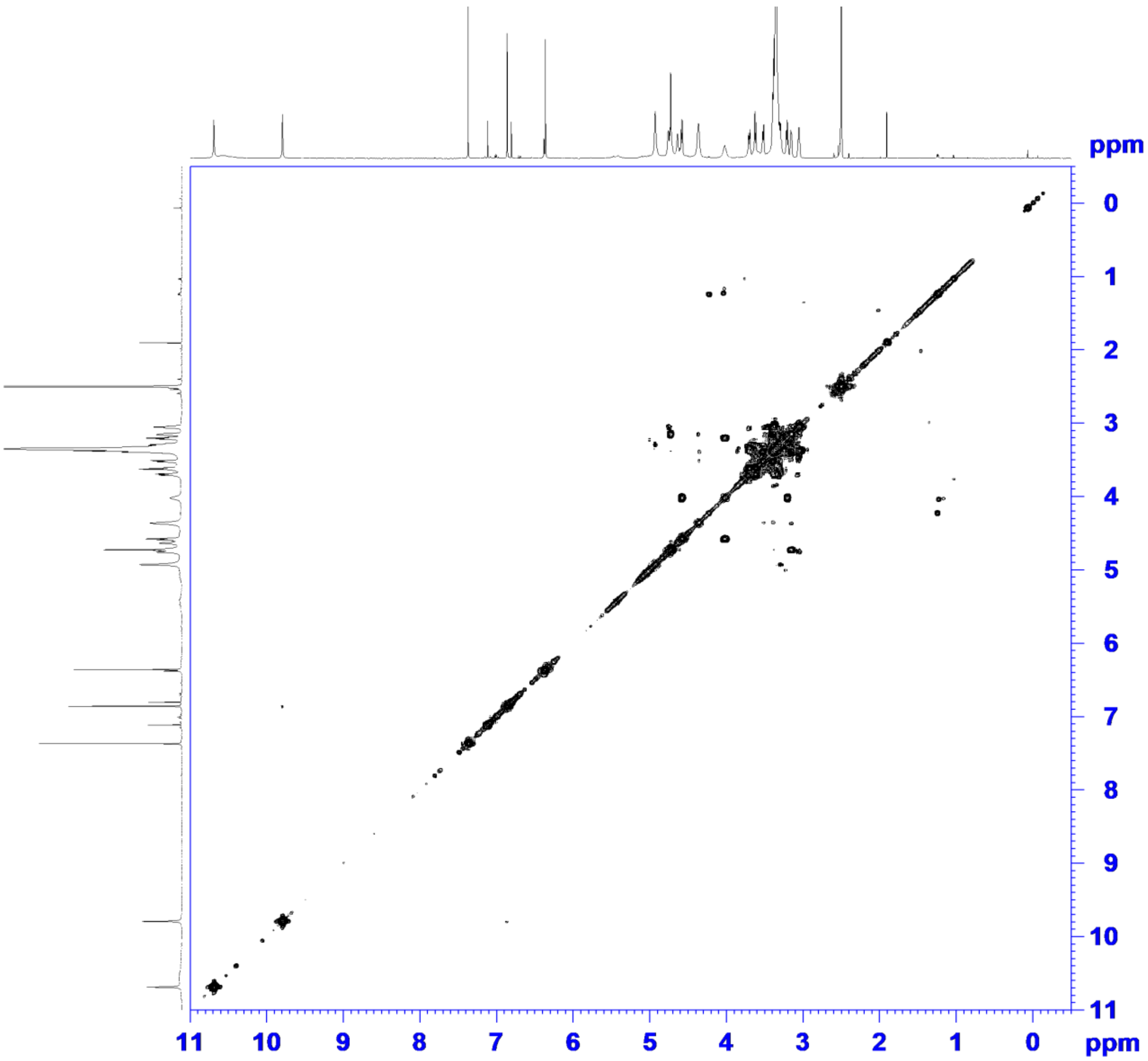

Figure S8. 2D NMR spectrum (^1H-^1H COSY, 700 MHz, DMSO-d_6) of the glucosyl-α-(1→6)-mangiferin (**1**).

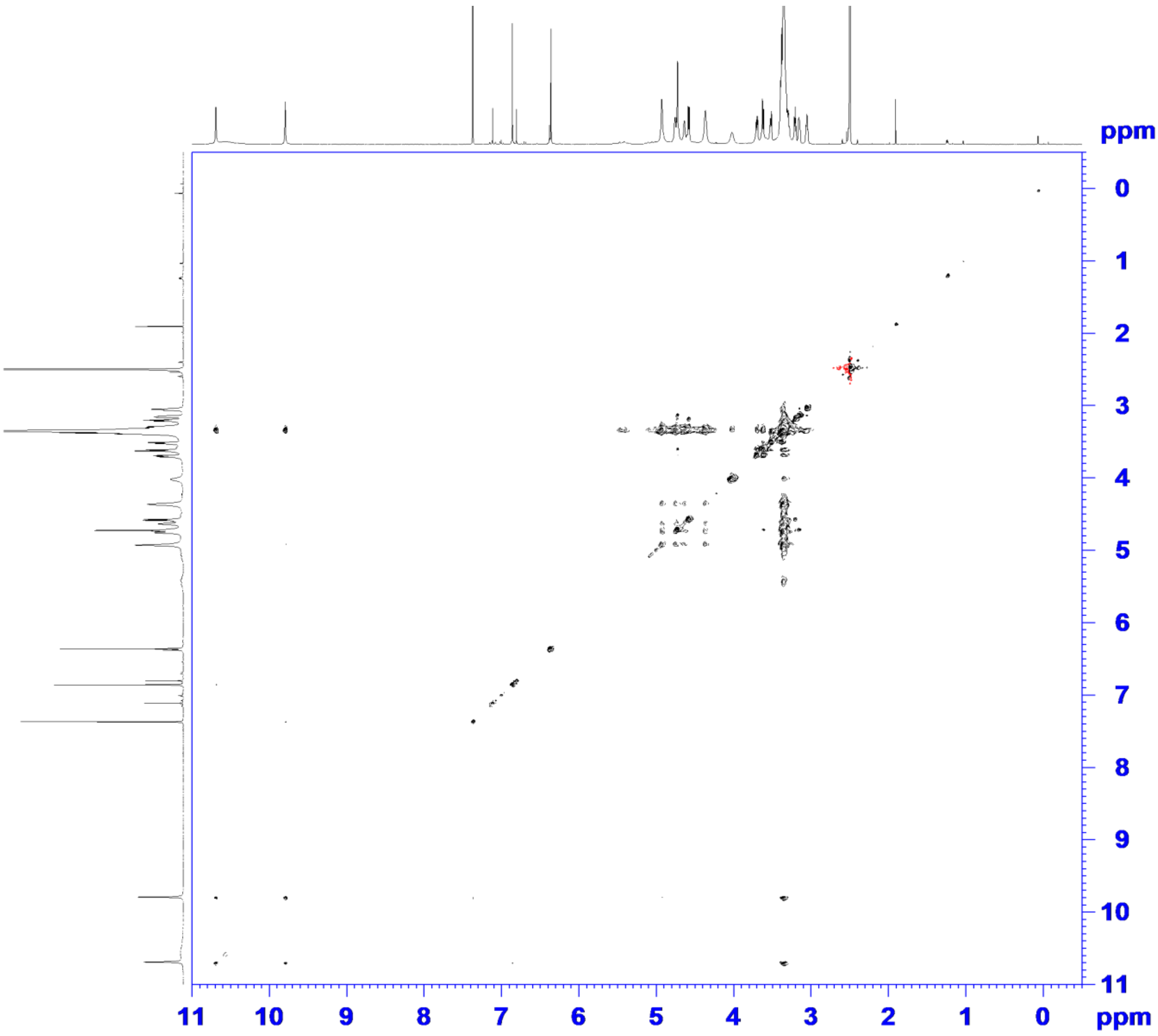

Figure S9. 2D NMR spectrum (^{1}H-^{1}H NOESY, 700 MHz, DMSO-d_6) of the glucosyl-α-(1→6)-mangiferin (**1**).

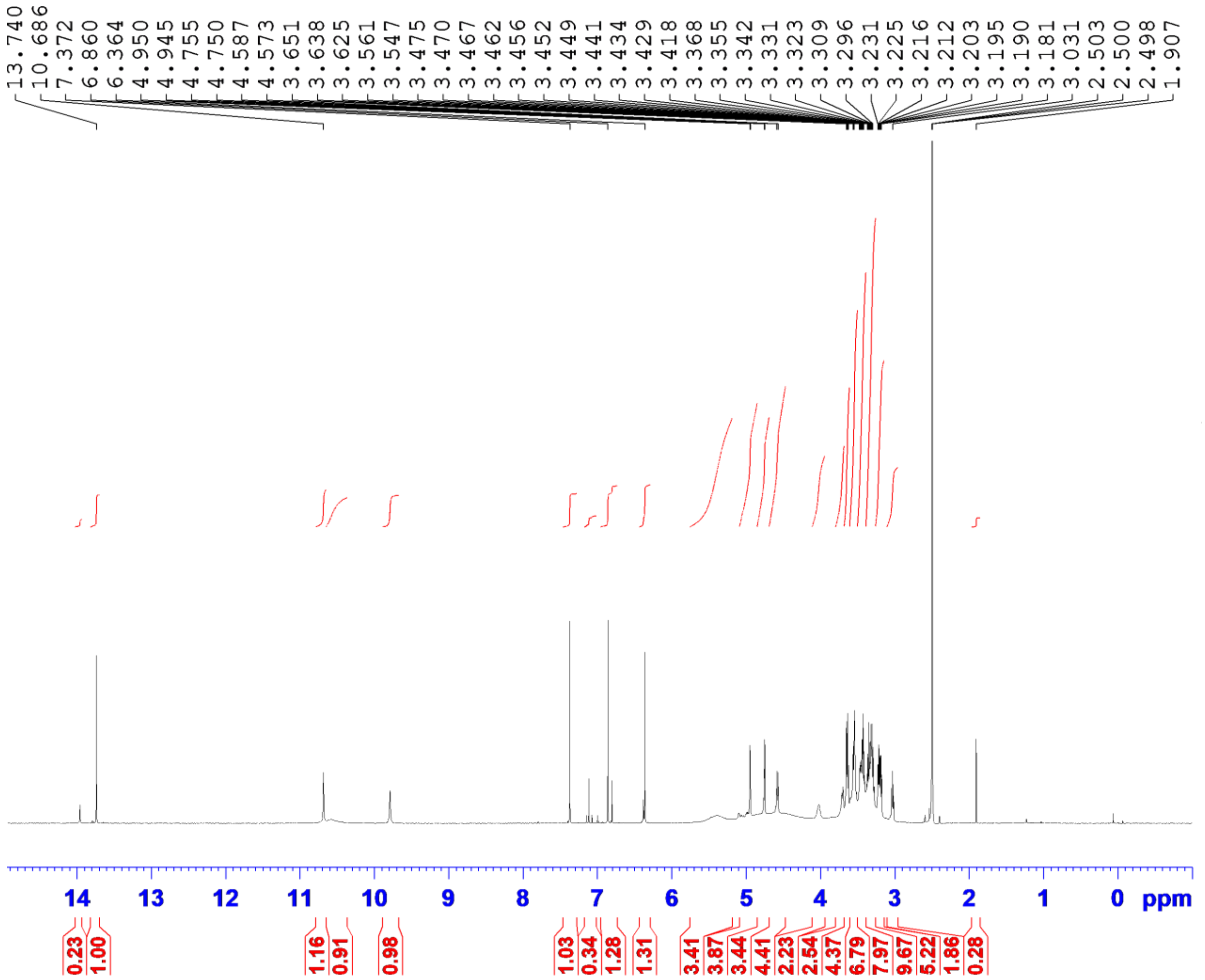

Figure S10. 1D NMR spectrum (^{1}H-NMR, 700 MHz, DMSO-d_6) of the maltosyl-α-(1→6)-mangiferin (**2**).

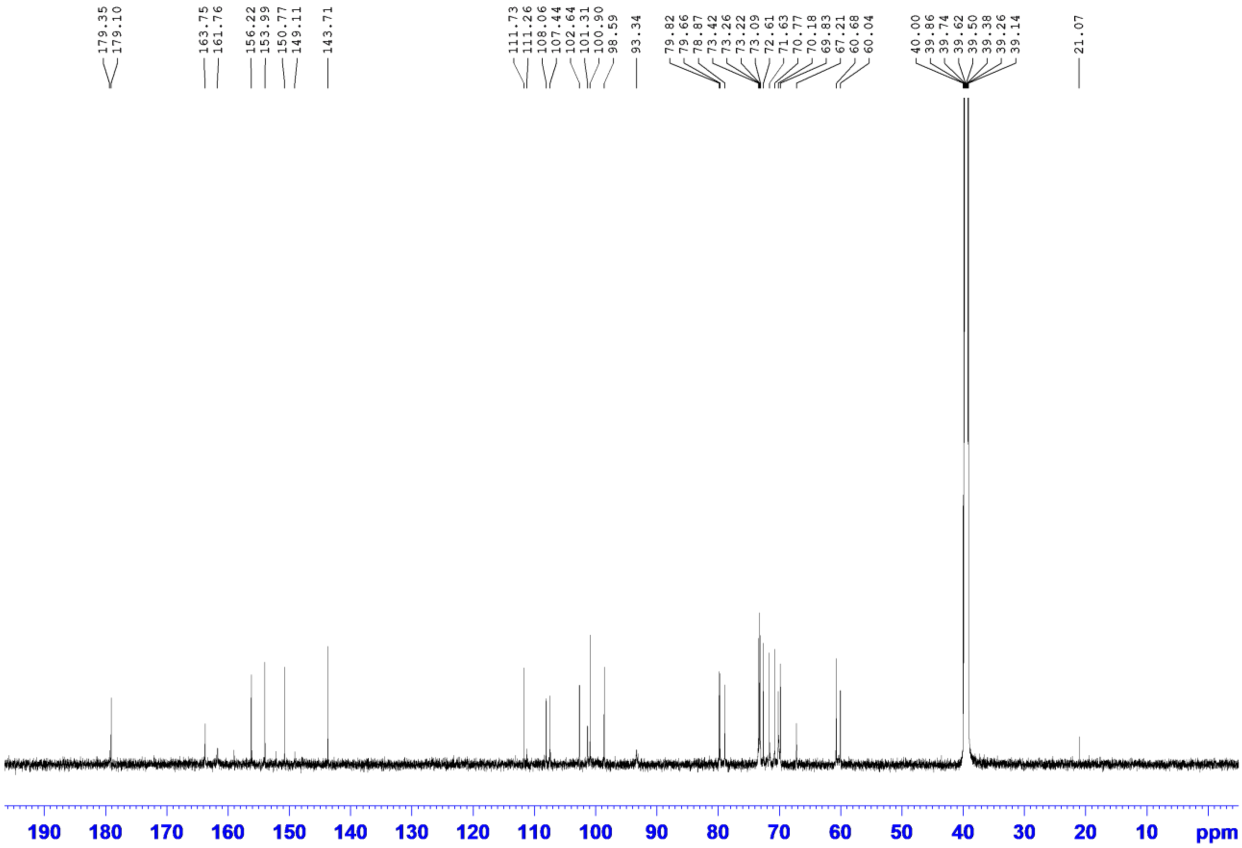

Figure S11. 1D NMR spectrum (^{13}C-NMR, 175 MHz, DMSO-d_6) of the maltosyl-α-(1→6)-mangiferin (**2**).

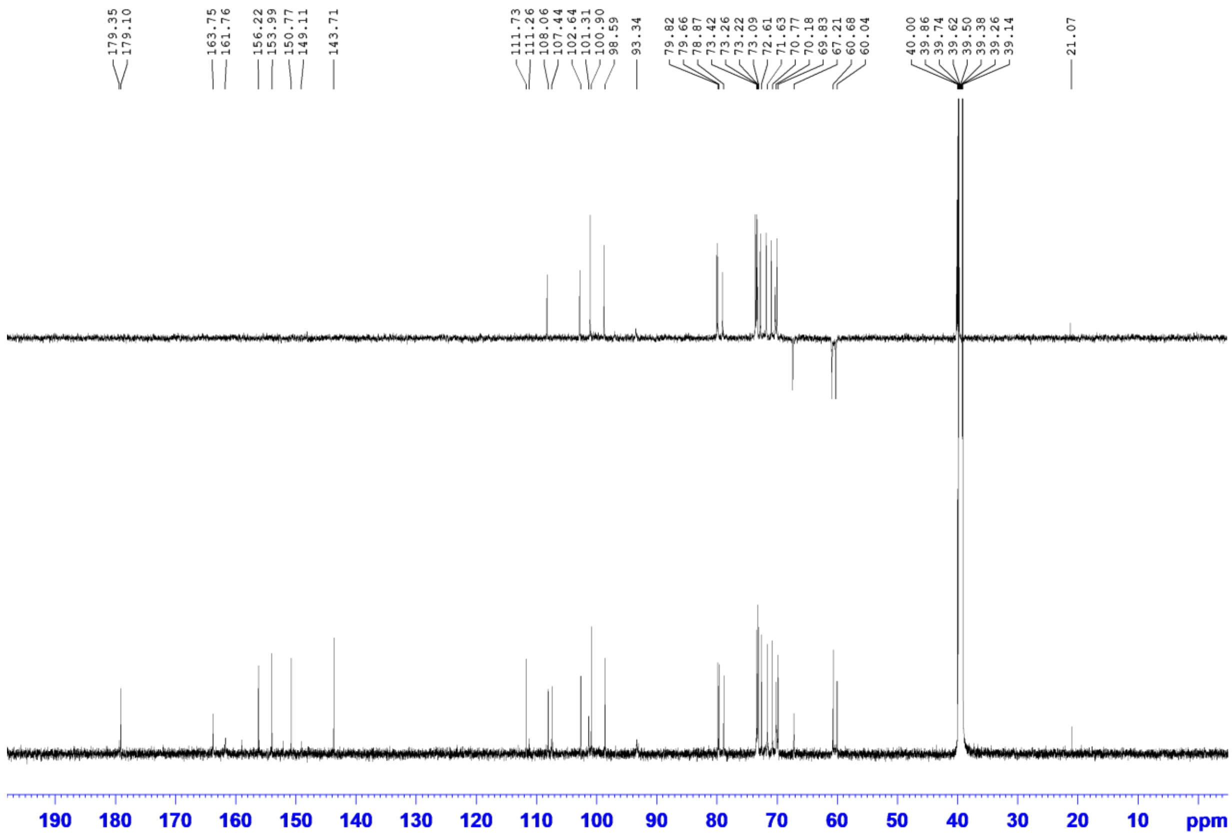

Figure S12. 1D NMR spectrum (DEPT-135, 175 MHz, DMSO-d_6) of the maltosyl-α-(1→6)-mangiferin (**2**).

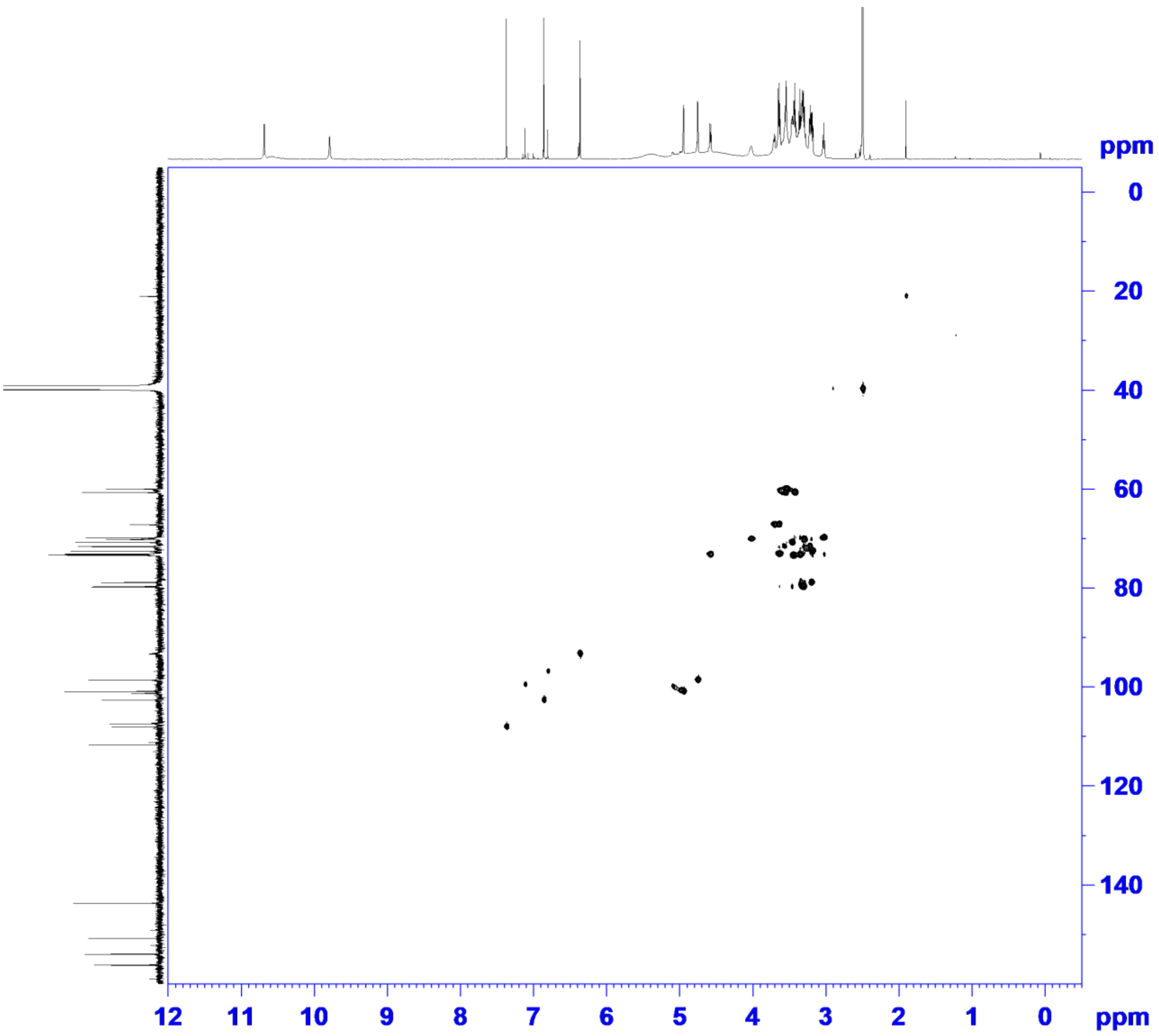

Figure S13. 2D NMR spectrum (^{1}H-^{13}C HSQC, 700 MHz, DMSO-d_6) of the maltosyl-α-(1→6)-mangiferin (**2**).

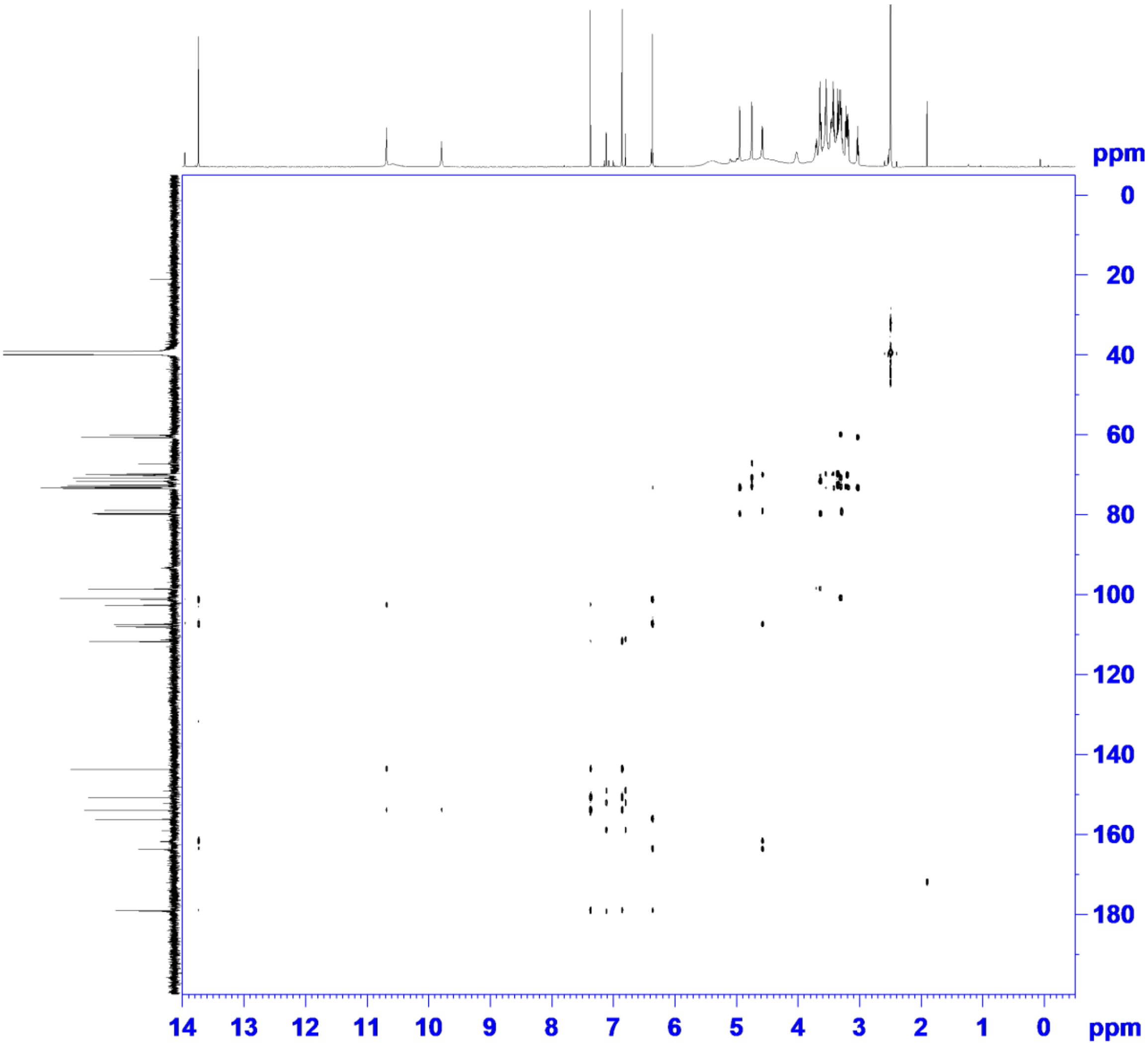

Figure S14. 2D NMR spectrum (^1H-^{13}C HMBC, 700 MHz, DMSO-d_6) of the maltosyl-α-(1→6)-mangiferin (**2**).

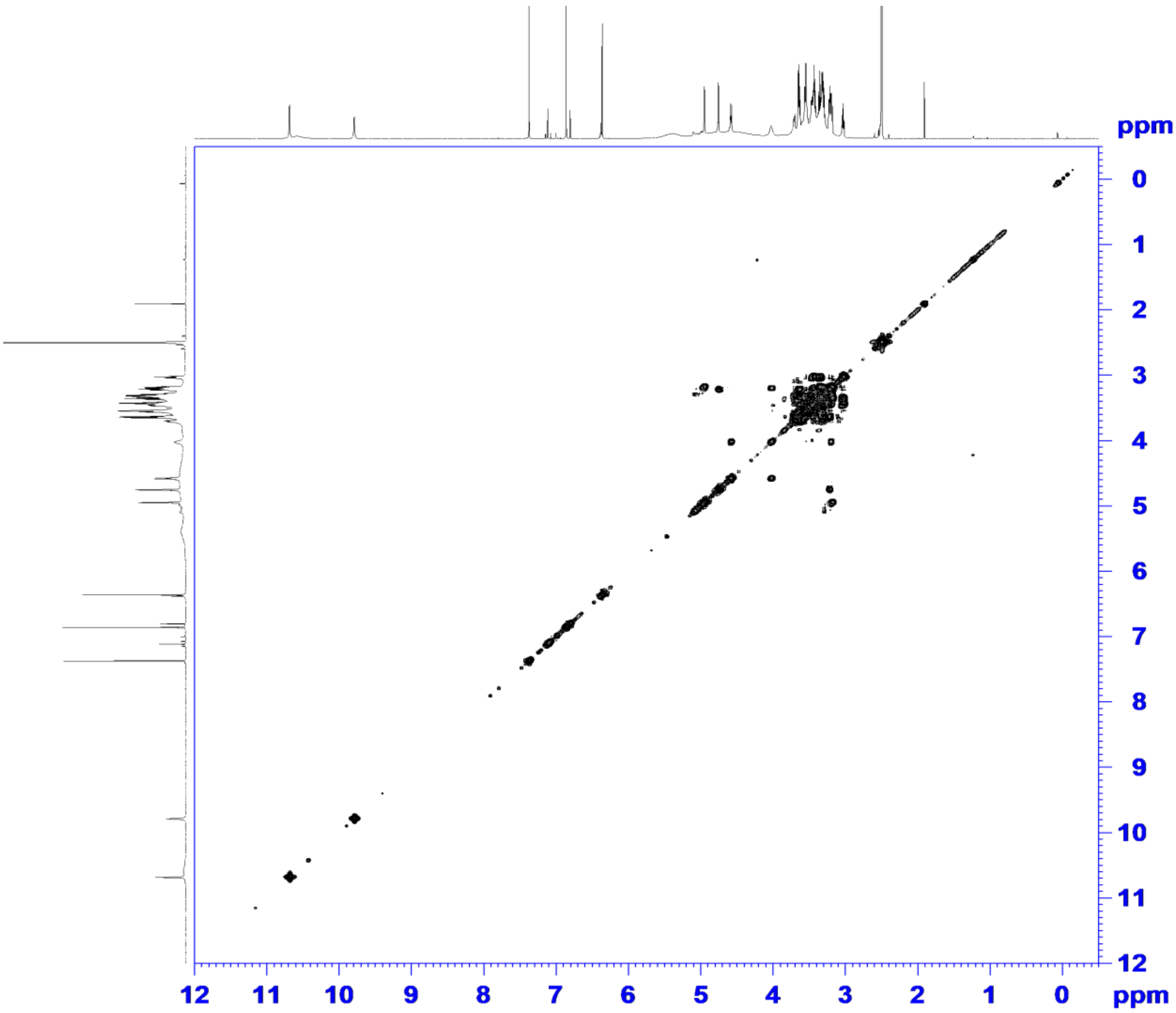

Figure S15. 2D NMR spectrum (^{1}H-^{1}H COSY, 700 MHz, DMSO-d_6) of the maltosyl-α-(1→6)-mangiferin (**2**).

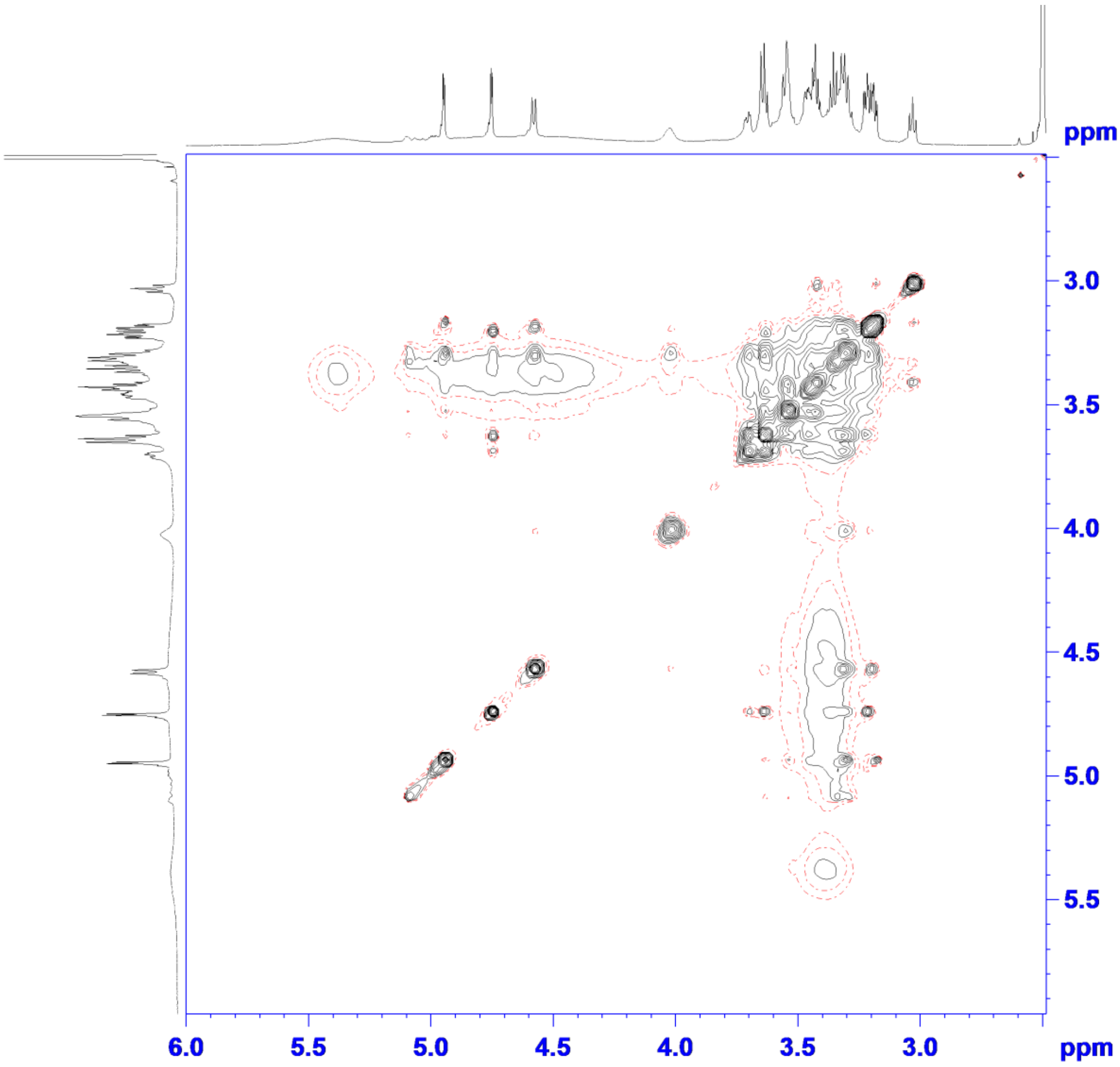

Figure S16. 2D NMR spectrum (^1H-^1H NOESY, 700 MHz, DMSO-d_6) of the maltosyl-α-(1→6)-mangiferin (**2**).
